# SLFN11 captures cancer-immunity interactions associated with platinum sensitivity in high-grade serous ovarian cancer

**DOI:** 10.1172/jci.insight.146098

**Published:** 2021-09-22

**Authors:** Claudia Winkler, Matthew King, Julie Berthe, Domenico Ferraioli, Anna Garuti, Federica Grillo, Jaime Rodriguez-Canales, Lorenzo Ferrando, Nicolas Chopin, Isabelle Ray-Coquard, Oona Delpuech, Davide Bedognetti, Alberto Ballestrero, Elisabetta Leo, Gabriele Zoppoli

**Affiliations:** 1 Bioscience and; 2 Translational Medicine, Oncology R&D, AstraZeneca, Cambridge, United Kingdom.; 3 Centre Léon Bérard, Lyon, France.; 4 Department of Internal Medicine and Medical Specialties and; 5 Department of Integrated Surgical and Diagnostic Sciences, University of Genova, Genova, Italy.; 6 IRCCS Ospedale Policlinico San Martino, Genova, Italy.; 7 Translational Medicine Pathology, Oncology R&D, AstraZeneca, Gaithersburg, Maryland, USA.; 8 Cancer Research Department, Sidra Medicine, Doha, Qatar.; 9 Hamad Bin Khalifa University, Doha, Qatar.

**Keywords:** Cell Biology, Oncology, Bioinformatics, Cancer, Cellular immune response

## Abstract

Large independent analyses on cancer cell lines followed by functional studies have identified Schlafen 11 (SLFN11), a putative helicase, as the strongest predictor of sensitivity to DNA-damaging agents (DDAs), including platinum. However, its role as a prognostic biomarker is undefined, partially due to the lack of validated methods to score SLFN11 in human tissues. Here, we implemented a pipeline to quantify SLFN11 in human cancer samples. By analyzing a cohort of high-grade serous ovarian carcinoma (HGSOC) specimens before platinum-based chemotherapy treatment, we show, for the first time to our knowledge, that SLFN11 density in both the neoplastic and microenvironmental components was independently associated with favorable outcome. We observed SLFN11 expression in both infiltrating innate and adaptive immune cells, and analyses in a second, independent, cohort revealed that SLFN11 was associated with immune activation in HGSOC. We found that platinum treatments activated immune-related pathways in ovarian cancer cells in an SLFN11-dependent manner, representative of tumor-immune transactivation. Moreover, SLFN11 expression was induced in activated, isolated immune cell subpopulations, hinting that SLFN11 in the immune compartment may be an indicator of immune transactivation. In summary, we propose SLFN11 is a dual biomarker capturing simultaneously interconnected immunological and cancer cell–intrinsic functional dispositions associated with sensitivity to DDA treatment.

## Graphical abstract

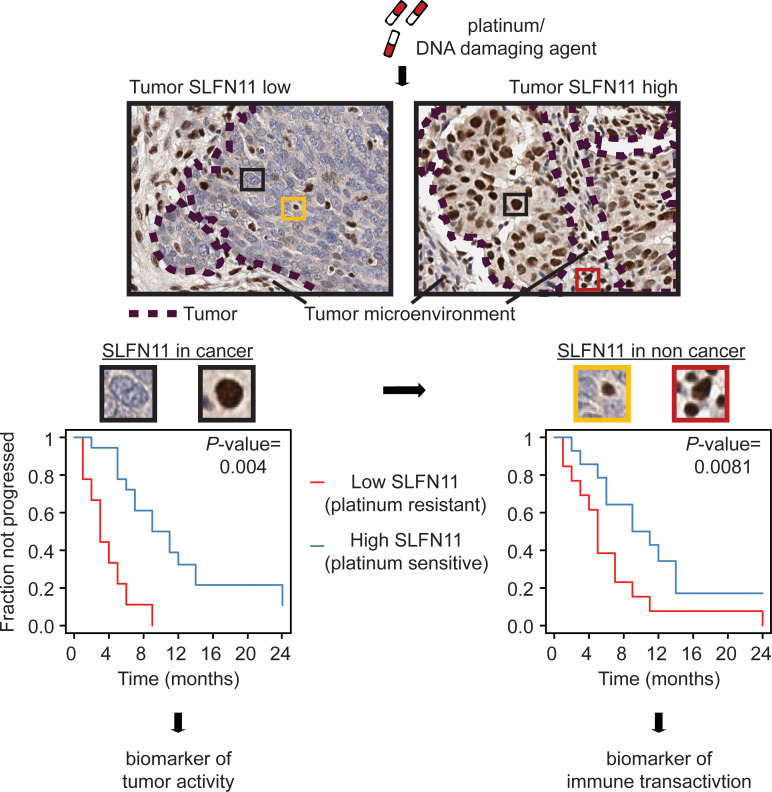

## Introduction

The putative DNA/RNA helicase Schlafen 11 (SLFN11) was independently reported by us (1) and others as the factor that best correlates with the response of cancer cells to DNA-damaging agents (DDAs) with different modes of action, such as topoisomerase I (e.g., topotecan and irinotecan) (2, 3), topoisomerase II inhibitors (e.g., epirubicin), and alkylating agents (e.g., cyclophosphamide or platinum) (4–6). Subsequently, a positive association between SLFN11 and sensitivity to Poly (ADP-ribose) polymerase (PARP) inhibitors was also described in preclinical models (6–9). After our discovery, several studies confirmed the causal role of SLFN11 in the process of cell death upon DDA treatment in cell lines (10, 11), organoids (5), and xenografts (3, 4, 6) from different tumor types. Moreover, SLFN11 has been recently studied in relation with the immune system (6, 12), especially in breast cancer (13), and for its potential role as an endogenous inhibitor of viral replication (14) and translation of DNA damage response proteins (15). Taken together, the available literature suggests that SLFN11 may play a so far incompletely understood role in an intertwined process of cancer and immune response to DDA-based chemotherapies. Indeed, it has been shown that SLFN11 is strictly correlated with immune-related transcripts in breast cancer (13), and its expression is regulated by IFN signaling in primary human cells (14, 16) as well as possibly in neoplastic cells (6, 12), hence pointing toward an exogenous regulation of its levels by the tumor-infiltrating immune milieu. One of the human cancers whose standard-of-care (SoC) treatment relies upon DDAs, and that is considered particularly sensitive to this category of chemotherapeutics, is high-grade serous ovarian carcinoma (HGSOC). HGSOC is the most common histologic subtype of ovarian cancer, accounting for three-quarters of newly diagnosed cases (17). Initial SoC treatment for advanced-stage HGSOC (the most frequent presentation stage for this poor-prognosis disease) consists of a platinum salt-taxane chemotherapy (CT) combination regimen, interposed or preceded by surgical debulking (18). In spite of macroscopically complete resection and upfront CT, most patients with HGSOC will eventually progress and die from their disease. In this context, several studies have shown that tumor-infiltrating lymphocytes (TILs), especially CD3^+^ and CD8^+^ TILs, may have a role as prognostic biomarkers, but their clinical utility is still unclear (19). The main aim of our study was to determine whether SLFN11 transcript and protein could be accurately and reproducibly measured in 2 different cohorts of serous ovarian cancer, one internal and another from The Cancer Genome Atlas (TCGA), considering the following aspects: (a) the sensitivity of HGSOC to DDAs, (b) the need for clinically useful prognostic biomarkers for CT treatments, (c) the potential connection between SLFN11 and TILs, and (d) the potential of developing SLFN11 as a biomarker in the clinic, based on results in preclinical models. We also aimed to gain mechanistic insights into the link between SLFN11 cancer immunity and DNA-damaging treatment, and most importantly, we investigated whether SLFN11 could represent a relevant prognostic biomarker to platinum-based treatment response in patients with advanced-stage HGSOC.

## Results

### Demographics.

The clinicopathological features of HGSOC cases selected for the present analysis, as detailed in the Methods section, are reported in Supplemental Table 1; supplemental material available online with this article; https://doi.org/10.1172/jci.insight.146098DS1 The proportions of patients with advanced-stage HGSOC were balanced between platinum-resistant (PR, *n* = 13) patients, defined as progressing within 6 months from the end of the last dose of platinum-based CT, and platinum-sensitive (PS, *n* = 15) ones (85% and 87%, respectively), as was the median number of completed cycles (7 in both groups). Median progression-free interval (PFI) was 4 months (95% CI = 2–6) in PR patients and 11 months (95% CI = 9–not reached) in PS ones. The patients had a median age of 62.4 years (95% CI = 56.8–66.9). In the studied cohort, PR patients were on average older than PS ones (65.7 vs. 58.2 years, *P* = 0.0026).

### SLFN11 levels are precisely defined by both transcript and protein levels in HGSOC samples.

**Figure 1:**
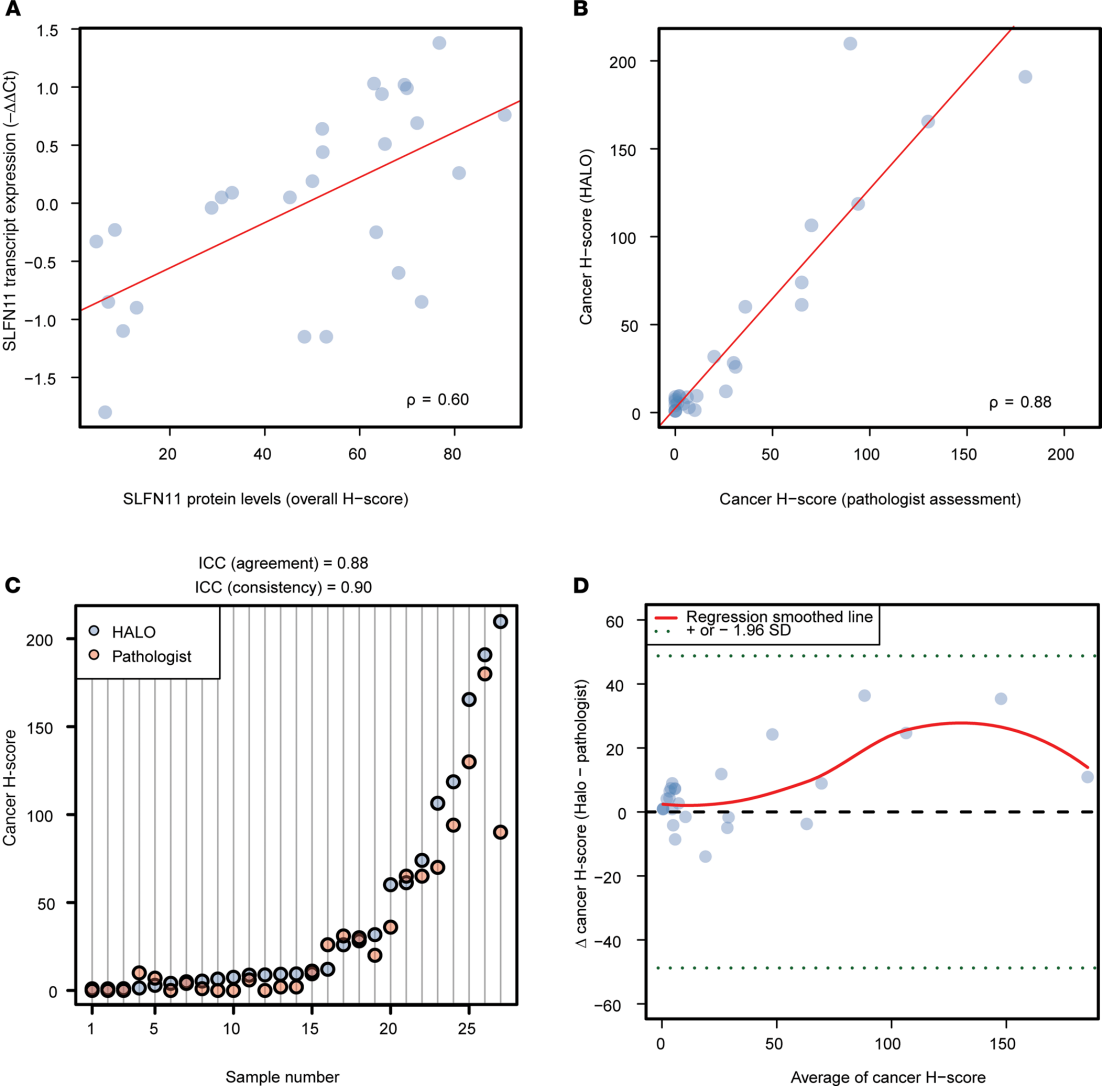
SLFN11 transcript and protein levels in HGSOC. (**A**) Scatterplot representing SLFN11 transcript by qRT-PCR as –ΔΔCt (*y* axis) as a function of its protein assessment by IHC as H-score (*x* axis) in the nucleus of noncancer and cancer cells from HGSOC specimens; ρ is the Spearman’s correlation coefficient; the least squares regression is represented by the red line; and dots are measurements of SLFN11 by qRT-PCR and IHC in individual samples. (**B**) Scatterplot representing SLFN11 protein levels in HGSOC cancer cells. *X* axis: pathologist’s assessment; *y* axis: H-score measured by HALO Digital Pathology (DP) software. (**C**) Dot plot illustrating cancer cell H-scores in individual samples (*y* axis), ordered by increasing DP-assigned values (*x* axis), highlighting the excellent consistency of intraclass correlation coefficients (ICCs) between the 2 methods. Each dot represents a score assigned by either the DP software (HALO) or the pathologist performing the assessment. (**D**) Bland-Altman plot displaying the difference between HALO’s and pathologist’s H-scores for cancer cells (*y* axis) by the increasing mean of value couples for individual samples (*x* axis). All points lie within 1.96 SDs (dotted green horizontal lines) from the mean difference (dashed horizontal black line), indicating no relevant bias between raters, and an insignificant trend toward higher H-scores for HALO as the mean values increase. The red line represents a smoothed regression (loess) fit of the actual mean scores.

To assess SLFN11 in HGSOC cases, we evaluated (a) the transcript levels by quantitative real-time polymerase chain reaction (qRT-PCR) as –ΔΔCt and (b) the protein levels in FFPE samples by IHC as H-score, blindly measured by the HALO image analysis platform (see Supplemental Table 2 and Supplemental Figure 1). Transcript and protein levels showed a strongly significant correlation (ρ = 0.52, *P* = 0.0051, respectively; see Figure 1A). This suggests that independent methods to measure SLFN11 in FFPE tissues lead to comparable results and that assessing SLFN11 transcript or protein levels are both acceptable to analytically quantify total SLFN11 in tissue.

We next sought to evaluate SLFN11 H-scores selectively in the cells from cancer tissue. Blind evaluation by our trained pathologist was found to highly correlate with the quantification obtained with the digital pathology software HALO (ρ = 0.88, *P* < 0.0001, respectively; see Figure 1B), with excellent reliability (20) (intraclass correlation coefficient [ICC] for agreement = 0.88 and ICC for consistency = 0.90; see Figure 1C), no relevant bias, and a very slight trend toward higher H-scores given by HALO for higher means, as evaluated with the Bland-Altman limits of agreement method (see Figure 1D). Taken together, these results established the analytical validity of our IHC approach to SLFN11 measurement in tumor specimens. Confidence in the automated digital pathology allowed us to launch an automated assessment of SLFN11 levels in either cancer or noncancer cells in each slide from the FFPE tissue samples.

### Total and intratumor infiltrating lymphocytes contribute to SLFN11 levels in HGSOC.

**Figure 2:**
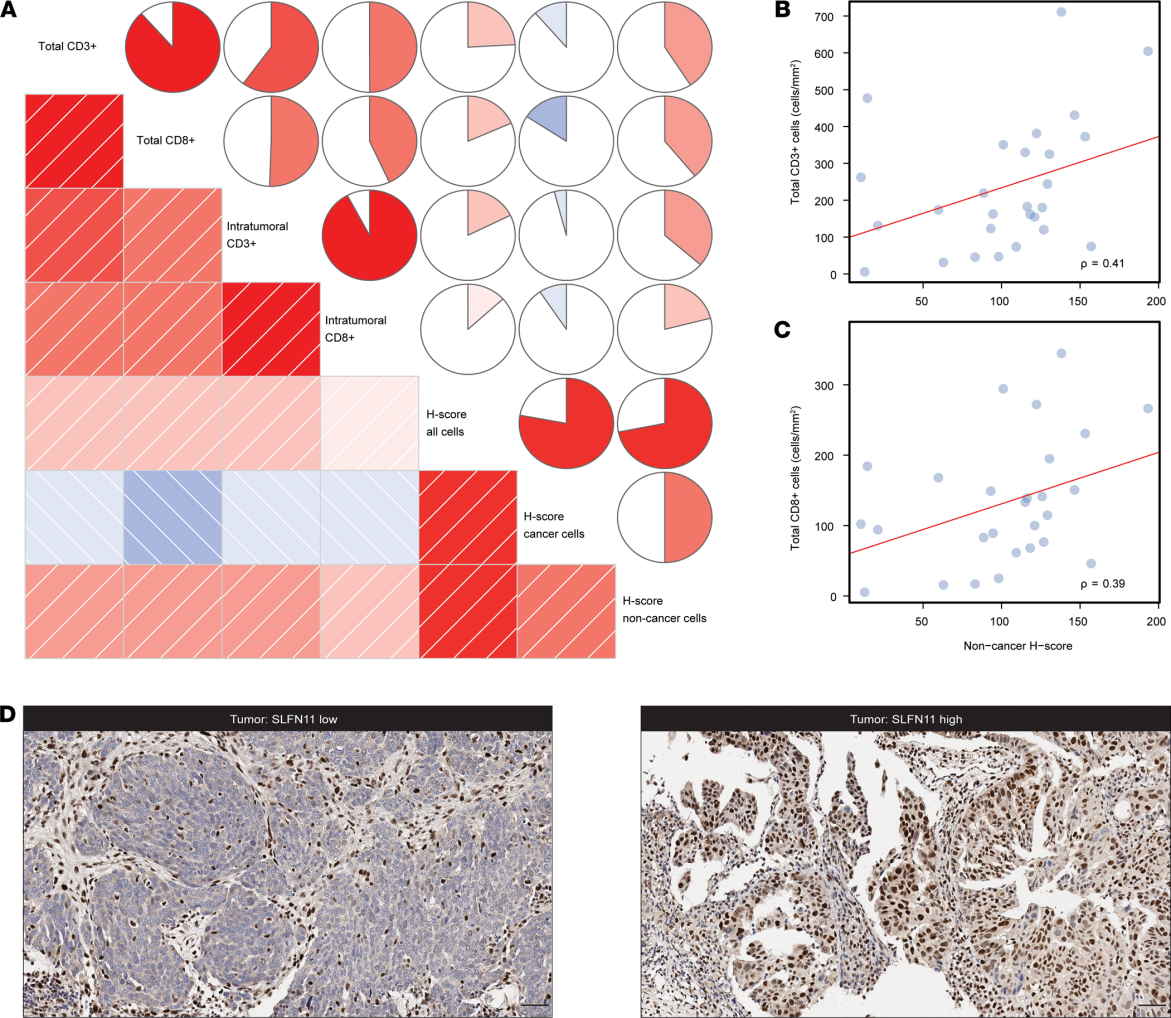
SLFN11 protein levels in HGSOC and their correlation with TILs. (**A**) Correlogram of TILs and SLFN11 H-scores, assessed in overall, cancer, and noncancer cells. In the lower triangle of the graph, boxes represent pairwise correlations colored by direction (blue for negative correlations and red for positive ones) and strength (intensity of shading) of the correlation itself. In the upper triangle, circles use the same scaled colors, but fill an area proportional to the absolute value of the correlation, and are filled clockwise for positive values, counterclockwise for negative values. (**B** and **C**) Scatterplots representing total CD3^+^ cells (**B**) and total CD8^+^ cells (**C**) — (*y* axes, cells/mm) as a function of SLFN11 H-score in noncancer cells (*x* axis); ρ is the Spearman’s correlation coefficient; the least squares regression are represented by the red lines, whereas dots are measurements of immune cell counts by H-scores in individual samples. (**D**) Representative images of SLFN11 IHC in HGSOC specimens. Left, stroma SLFN11^hi^ and tumor SLFN11^lo^; right, stroma and tumor SLFN11^hi^, for the indicated cancers. The images highlight nuclear SLFN11 protein localization in tumor cells and different stromal cell subtypes. Scale bars: 50 μm.

Since several studies have reported on the role of TILs in the prognosis of ovarian cancer (21–24), and SLFN11 has been shown to be expressed in primary human T lymphocytes (16), we evaluated TIL infiltration by CD3 and CD8 staining, both in terms of total number (total CD3^+^ and CD8^+^ TILs) and of a measure of TILs in direct contact with cancer cells, without stroma interposition (intratumor CD3^+^ and CD8^+^ TILs) in HGSOC (19). Using HALO, we then calculated SLFN11 overall H-scores in the studied cohort, as well as H-scores in cancer and noncancer cells separately (see Supplemental Table 2). H-scores measured in noncancer cells showed the strongest correlations with TILs, whereas the H-scores in cancer cells exhibited a nonsignificant negative association with TIL counts (see Figure 2A and Supplemental Table 3). In particular, noncancer H-score correlations with total CD3^+^ and CD8^+^ TILs were moderate at 0.41 (FDR = 0.0723, see Figure 2B) and 0.39 (FDR = 0.0852, see Figure 2C), respectively. Indeed, SLFN11 protein assessment in the clinical specimens revealed its localization in both cancer cells and stromal cells of various origins (see Figure 2D for representative images of stroma SLFN11^hi^ and tumor SLFN11^hi^ or SLFN11^lo^). Taken together, these results indicate that, in addition to TILs, other cell populations contribute to SLFN11 protein levels in tumor tissues. Of interest, a moderate association between cancer and noncancer SLFN11 levels could be observed (ρ = 0.50, FDR = 0.0208).

### SLFN11 in cancer and noncancer cells independently predicts response to platinum-based CT in HGSOC.

**Figure 3:**
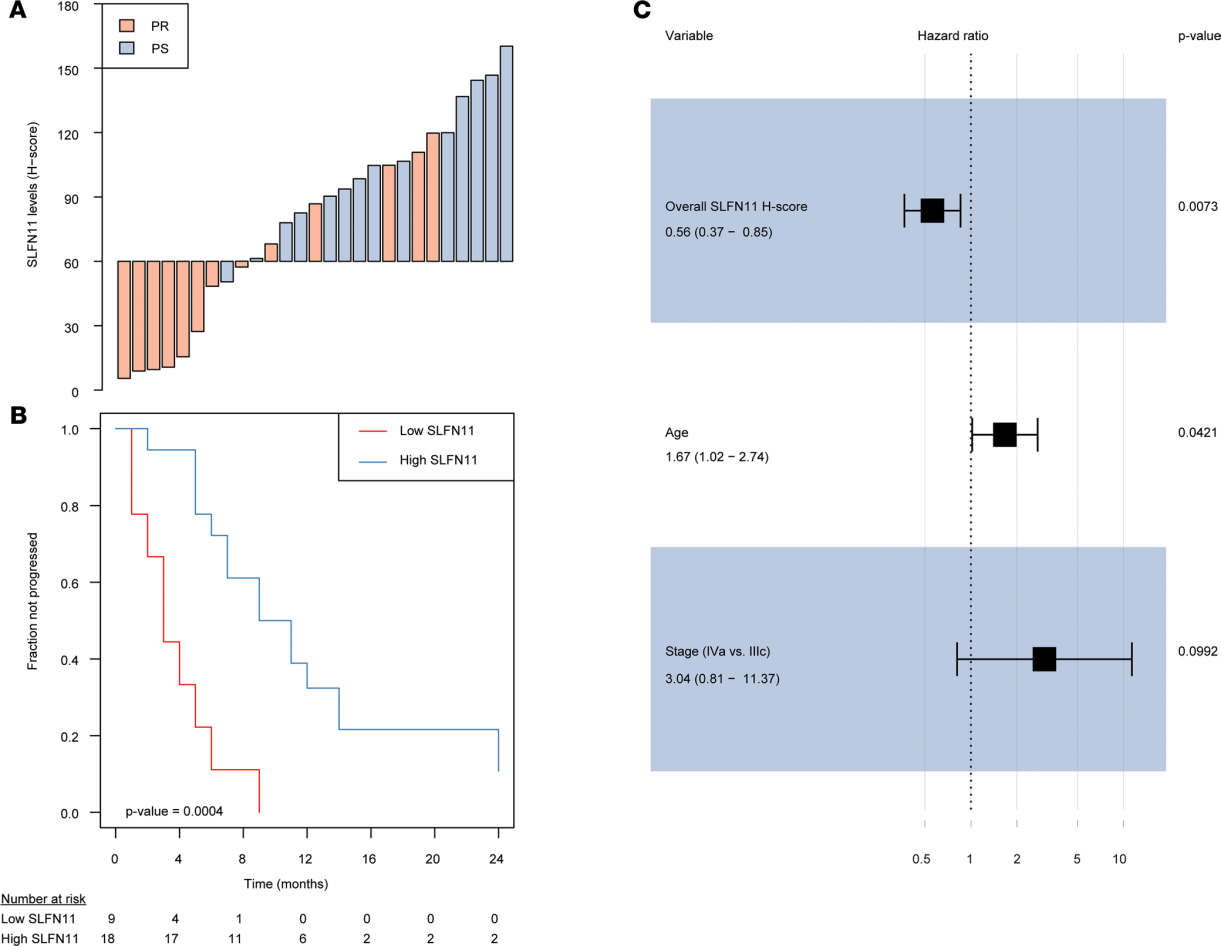
SLFN11 protein levels measured in cancer and noncancer cells are independently prognostic in HGSOC. (**A**) Waterfall plot showing SLFN11 overall protein levels (i.e., measured in cancer and noncancer cells) in individual cases, colored by platinum sensitivity: SLFN11 protein is reported as H-score (*y* axis), whereas cases are reported by increasing values (*x* axis) and colored in red if platinum refractory (PR) or light blue if nonrefractory (NR). (**B**) Kaplan-Meier plot showing PFI stratified by SLFN11 overall protein levels (“high” if H-score > 60, “low” if < 60). The progressed fraction of patients (*y* axis) is plotted against time expressed in months from the end of first-line CT, censored at 24 months (*x* axis). Numbers at risk are reported below the plot. *P* value in the bottom left of the plot is from the Wald statistics for the univariable Cox’s regression. (**C**) Forest plot of HRs (*x* axis, in log scale) for variables retained in the final multiple Cox’s regression model. Point HR estimates are reported below each variable together with 95% CIs in parentheses, whereas adjusted *P* values for each variable are on the right side of the plot. Filled black squares represent HR estimates, with relative 95% CI shown as horizontal lines with brackets.

We next sought to explore whether higher SLFN11 protein levels associate with better outcome in the platinum-treated advanced stage in our HGSOC cohort. First, we evaluated the impact of SLFN11 overall H-score, H-score in cancer and noncancer cells, stage, age, and TIL infiltration on PFI by univariable statistics. Overall and noncancer higher SLFN11 H-scores were strongly associated with a better prognosis (HR = 0.50, 95% CI = 0.33–0.75, *P* = 0.0009; and HR = 0.54, 95% CI = 0.36–0.81, *P* = 0.0028, respectively). Among the other variables with possible prognostic impact assessed in our cohort, older age was associated with shorter PFI (HR = 1.83, 95% CI = 1.19–2.82, *P* = 0.0062), whereas higher total CD3^+^ TILs were associated with longer PFI (HR = 0.55, 95% CI = 0.34–0.90, *P* = 0.0180). A borderline significant association with shorter PFI was observed for stage IVa versus IIIc cancer (HR = 3.19, 95% CI = 0.89–11.46, *P* = 0.0758), whereas an opposite trend was found for higher total CD8^+^ TIL count (HR = 0.67, 95% CI = 0.41–1.09, *P* = 0.1040) and SLFN11 H-score assessed in cancer cells only (HR = 0.62, 95% CI = 0.38–1.02, *P* = 0.0620). With an H-score cutoff of 60, obtained by maximizing the accuracy to classify PR versus PS cases in our cohort, overall SLFN11 protein levels had an accuracy of 0.78, with sensitivity of 0.93 and specificity of 0.62 (see Figure 3A). The association of overall SLFN11 H-score as a binary variable with PFI was indeed significant, with an HR = 0.17 (95% CI 0.06–0.45, *P* = 0.0004; see Figure 3B). When the most significant measure of SLFN11, i.e., the overall H-score, and the other variables with a *P* value no more than 0.1 were entered in a stepwise forward-backward multivariable Cox’s regression model, overall SLFN11 protein levels retained their independent prognostic value (adjusted HR = 0.56, 95% CI = 0.37–0.85, *P* = 0.0073), together with age and stage (see Figure 3C). Albeit exploratory in nature, these results were surprising in several regards. First, the independent prognostic value of overall SLFN11 H-score suggests that SLFN11 levels in both cancer and noncancer cells may play a role in response to platinum-containing regimens in HGSOC. Indeed, dichotomized SLFN11 cancer (see Supplemental Figure 2, A and B) and noncancer (see Supplemental Figure 2, C and D) levels were also prognostic by univariable analysis (though with smaller significance than overall SLFN11, see Supplemental Table 4). Moreover, both cancer and noncancer SLFN11 retained their independent role in stepwise multivariable models starting from the same set of variables as the one including overall SLFN11 (see Supplemental Figure 2, E and F, and Supplemental Table 4). An interesting finding is that, when overall SLFN11 or noncancer SLFN11 were considered together with the other covariates to generate a multivariable model, CD3^+^ TILs lost their independent prognostic role. On the other hand, when cancer SLFN11 H-score was used to generate a multivariable model, that measure was independently significant together with CD3^+^ TILs (see Supplemental Figure 2E). This observation strengthens the hypothesis that cancer-expressed SLFN11 is directly linked with neoplastic cell sensitivity to DDAs like platinum independently of immune infiltration, whereas the prognostic relevance of noncancer SLFN11 may underlie an “active” tumor immune milieu. In turn, these findings would explain why overall SLFN11 H-score is a stronger prognostic biomarker than either cancer or noncancer SLFN11 measured separately.

### SLFN11 is expressed by cells of the innate and adaptive immune system infiltrating HGSOC.

**Figure 4:**
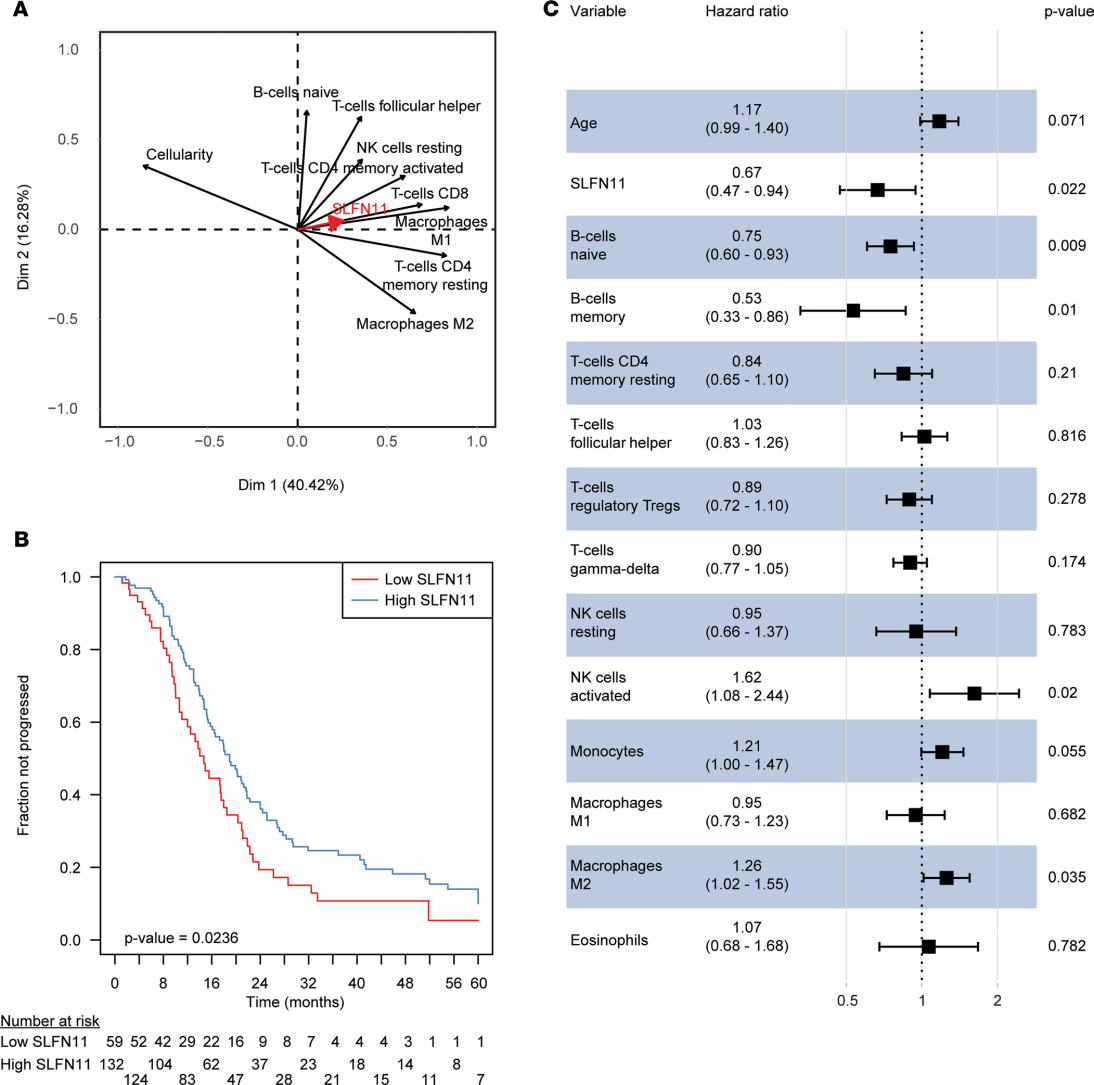
SLFN11, immune cell subpopulations, and prognosis in TCGA serous ovarian carcinoma data set. (**A**) Variable correlation plot of the PCA including SLFN11 transcript, cancer cellularity, and CIBERSORTx immune cell subpopulations significant by univariable correlation at FDR < 0.05. The 2 axes represent the first principal components explaining the greatest fraction of the variance of the analyzed data set, with percentage of explained variability in parentheses. The relative position of the variables toward each other explains their relative correlation, whereas their distance from the intersect accounts for their contribution to the components. SLFN11 is represented with a thick red arrow for sake of clarity. (**B**) Kaplan-Meier plot showing PFI stratified by SLFN11 transcript (“high” if above the lower tertile of expression in the data set, “low” if below). The progressed fraction of patients (*y* axis) is plotted against time expressed in months from the end of first-line CT, censored at 60 months (*x* axis). Numbers at risk are reported below the plot. *P* value in the bottom left of the plot is from the Wald statistics for the univariable Cox’s regression. (**C**) Forest plot of hazard ratios (*x* axis, in log scale) for variables retained in the lasso-selected multiple Cox’s regression model. Point HR estimates are reported below each variable together with 95% CIs in parentheses, whereas adjusted *P* values for each variable are on the right side of the plot. Filled black squares represent HR estimates, with relative 95% CIs shown as horizontal lines with brackets.

As our results indicated, SLFN11 was associated with the response to platinum-based treatment in HGSOC, due to its expression in both cancer and noncancer (immune-related) cells. Moreover, noncancer SLFN11 H-score showed only a moderate correlation with TILs, with both variables retaining an independent prognostic value in HGSOC. This suggests that other cell subpopulations contribute to the overall levels of SLFN11 in tissues. Hence, we sought to better define these populations. With this aim, we estimated cancer cellularity in a second HGSOC cohort from TCGA (*n* = 302 cases) with ESTIMATE (25), and we inferred leukocyte subpopulations using CIBERSORTx, a well-established method for characterizing the immune cell composition of tissues from their gene expression profiles (26). We first correlated the obtained values with SLFN11 transcript levels (see Supplemental Table 5). Of interest, not only adaptive immune system cells (CD4^+^, CD8^+^ T cells as well as B cells), but also macrophages and NK cells, showed a significant association with SLFN11 (FDR < 0.05). In fact, among immune cell subpopulations, macrophages showed the strictest correlations with SLFN11, and this observation was transversely confirmed for validation purposes by single-sample gene set enrichment analysis (correlation between enrichment score for macrophages and SLFN11 = 0.27, *P* < 0.0001; Supplemental Figure 3A). SLFN11 only marginally correlated with regulatory T cells (ρ = 0.13, FDR = 0.57; Supplemental Table 5). Moreover, publicly available RNA-sequencing data from sorted leukocyte subpopulations (Gene Expression Omnibus [GEO] accession GSE60420) corroborated our in silico findings: higher SLFN11 levels were observed in monocytes, followed by NK cells, CD8^+^ T cells, B cells, and CD4^+^ T cells, whereas — as in our results — the lowest SLFN11 transcript could be observed in neutrophils (see Supplemental Figure 3B). Cancer cellularity showed a negative correlation with SLFN11 expression in TCGA HGSOC set (ρ = –0.30, FDR < 0.0001; see Supplemental Figure 4A). This observation, independently confirmed in our cohort (ρ** = –0.42, *P* = 0.0276, see Supplemental Figure 4B), could be explained by hypothesizing that HGSOC cases with lower cancer cellularity would, in general, have higher tumor immune cell infiltration, with consequently higher SLFN11 levels. To explore this hypothesis, we performed principal component analysis (PCA), including cancer cellularity together with immune cell subpopulations correlating with SLFN11 with FDR < 0.05 (see Figure 4A). PCA is a dimensionality reduction method enabling the identification and visualization of correlations and patterns between variables without aprioristic assumptions about their mutual relationships. As anticipated, all immune subpopulations were represented as lying in opposition to cancer cellularity, in particular M1 macrophages, CD4^+^ memory resting T cells, and CD8^+^ T cells. Moreover, the SLFN11 expression vector was lying close to the vectors of the immune subpopulations it is more closely correlated to, such as T cells and macrophages. Overall, PCA substantiated our hypothesis that high immune infiltration was driving the negative correlation of SLFN11 with cancer cellularity and that specific immune subpopulations were closely associated with high SLFN11 expression in HGSOC.

### SLFN11 is independently prognostic in TCGA HGSOC data set.

Subsequently, we validated the prognostic role of SLFN11 in TCGA HGSOC patients. To do so, we selected stage IIIc/IV cases with histological grade 3 and at least 28 days of PFI (221 cases with 157 progression events). SLFN11 was associated with PFI (HR = 0.68, 95% CI = 0.49–0.95, *P* = 0.0233; see Figure 4B) and remained independently significant (adjusted HR = 0.67, 95% CI = 0.47–0.94, *P* = 0.0222), together with age and specific immune cell subpopulations, in a multivariable Cox’s proportional hazards model with variables selected by lasso regularization (see Figure 4C). Of relevance, B and T cell subpopulations were independently prognostic of an extended PFI, whereas monocytes, M2 (but not M1) macrophages, and activated NK cells were associated with poorer prognosis (for univariable survival analyses of immune cell subpopulation in TCGA data set, see Supplemental Table 6). This result is in line with SLFN11 correlations with immune cell subpopulations, in that B cells and NK resting cells were associated with higher SLFN11 transcript, while NK activated cells were not. The surprisingly negative association between NK and prognosis might be related to imbalance of different NK cell subsets associated with diverse immune-modulatory properties. Monocytes and macrophages exhibited heterogeneous behavior in regard with prognosis when assessed in a multivariable fashion. We did not, however, try to model the interactions of SLFN11 with those subpopulations, due to the interpretational complexity of results in the absence of functional experiments, as well as the limited numerosity of TCGA HGSOC data set for such a purpose. Likewise, we wished to avoid the overinterpretation of an exploratory analysis, which would, anyway, be derived from in silico deconvolution methods not devoid of the potential for error propagation.

### SLFN11 protein localization with immune cell subpopulations is supported in tonsil and HGSOC tissues.

**Figure 5:**
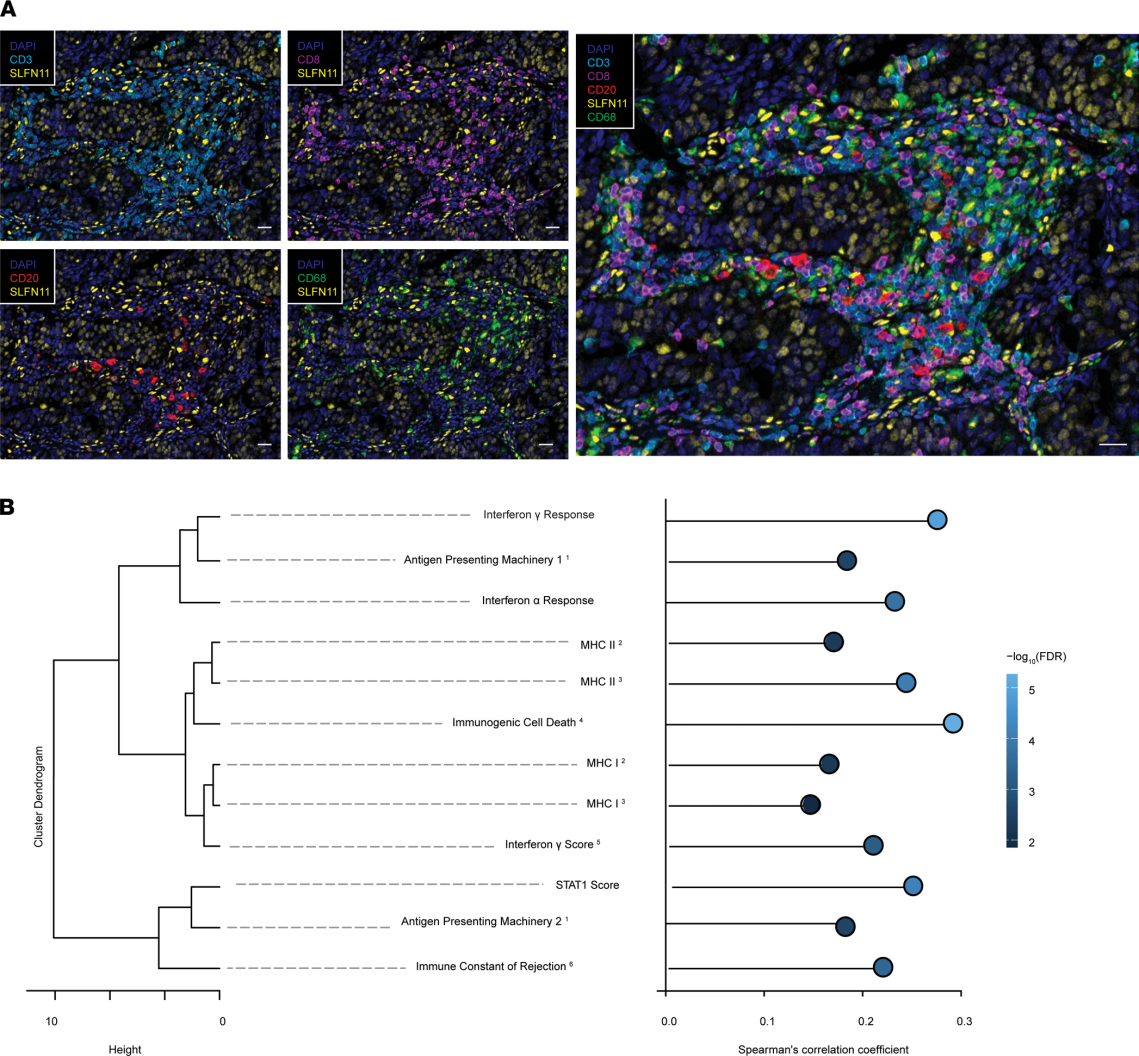
SLFN11 is expressed in a subset of immune-related cells and is associated with immune signatures in HGSOC. (**A**) Multiplex immunofluorescence for SLFN11 in T cells (CD3^+^CD8^+^), B cells (CD20^+^), and macrophages/monocytes (CD68^+^) infiltrating HGSOC tissue. Scale bars: 50 μm. (**B**) Dendrogram representing the similarity between different immunologic signatures calculated in TCGA ovarian cancer data set (*n* = 302 cases). *X* axis represents the Ward’s D2 distance. Signature names have superscript numbers to denote the publication they are derived from: 1: (32); 2: (31); 3: (30); 4: (38); 5: (28); if not specified, signatures are from 6: (27). Right: lollipop plot of correlations between SLFN11 and the aforementioned signatures. *X* axis represents the Spearman’s correlation coefficient between SLFN11 expression in TCGA ovarian cancer data set and the investigated signatures, whereas individual dots are colored by –log_10_ of the FDR of the correlation.

SLFN11 protein localization in a subset of cells in the immune cell compartment could be confirmed in tonsil and HGSOC (Supplemental Figure 5) tissues from our cohort. In serial sections of the tonsil, SLFN11-positive cells could be mainly found in the germinal center and the paracortical zone, and less in the mantle zone, of the lymphoid follicle. The germinal center was enriched in naive and memory B cells (CD20^+^) and monocytes/macrophages (CD68^+^), whereas the paracortical zone was mostly composed of T cells (e.g., CD3^+^ and CD8^+^) (Supplemental Figure 5A). In serial sections of HGSOC tissues, we could observe high SLFN11 protein in those immune cell subtypes, particularly in monocytes/macrophages (Supplemental Figure 5, B and C, for representative HGSOCs with varied SLFN11 protein in cancer cells). However, we also noted SLFN11 in other, to-be-defined, stromal cell subtypes. Finally, with multiplex IHC staining and multispectral image acquisition, we could support SLFN11 expression in infiltrating CD3^+^/CD8^+^ TILs, B cells, and macrophages (Figure 5A). Taken together, we demonstrate that SLFN11 is expressed in macrophages, TILs, and B cells but also in other to-be-defined stromal cell types in HGSOC.

### High SLFN11 expression is associated with immune activity signatures in HGSOC.

After demonstrating, in silico and through IHC analysis on an independent case cohort, that SLFN11 is expressed not only in cancer cells, but also in TILs, in macrophages, and in other immune cell subpopulations, we wondered how SLFN11 expression in HGSOC is correlated with biologically selected, well-established immune signatures representing hallmarks of immune activity, such as IFN-α and -γ signaling and STAT1 activation (27–29), MHC I and MHC II upregulation (30, 31), antigen presenting machinery (32), immunologic constant of rejection (33–37), and immunogenic cell death (38). To do so, we correlated SLFN11 with those signatures in the HGSOC TCGA RNA-sequencing data set (*n* = 302 cases). All the aforementioned signatures showed extremely significant positive correlations with SLFN11 expression, with close reproducibility among signatures of similar meaning generated by different authors (see Figure 5B). In particular, the 2 top correlating signatures with SLFN11 in HGSOC were the immunogenic cell death signature and the IFN-γ response hallmark signature. Taken together, these findings are suggestive of a close, hitherto scarcely investigated link between SLFN11 and cancer immunity in HGSOC.

### SLFN11 is a dual biomarker in HGSOC.

We next sought to investigate the mechanism by which high SLFN11 is associated with immune activation in HGSOC. Recently, it has been reported that SLFN11 in cancer cells activates an innate immune stress response following DDA treatment and replication stress (11). To further investigate such a potential link between SLFN11 cancer immunity and DDA treatment, we performed total RNA sequencing of SLFN11-proficient (DU145 wild type, WT) and SLFN11-deficient (DU145 SLFN11 knockout, KO) cells treated for 0, 6, or 24 hours with gemcitabine, a DNA synthesis inhibitor that acts primarily during the S-phase of the cell cycle and induces replication stress (39). Gemcitabine treatment induced striking transcriptomic differences in WT versus KO cells (2 different KO clones, Supplemental Figure 6A). A meta-analysis of the different comparisons (FDR < 0.01 and a cutoff of 2-fold change) identified 1089 upregulated and 459 downregulated genes (Supplemental Figure 6B and Supplemental Table 7). Interestingly, we found an enrichment of immune-regulatory pathways within the upregulated gene cluster (Supplemental Figure 6B), such as type II IFN-γ (IFNG), TNF-α, or the IL-18 signaling pathway (40). Remarkably, this was accompanied with downregulation of oncogenic signaling classically associated with immune exclusion, such as WNT and MAPK signaling (34, 41, 42). A total of 34 genes were differentially expressed in the IL-18 signaling pathway, and about 70% of these genes were enriched in the gemcitabine-treated versus untreated samples (Supplemental Figure 6C). Thereby, inflammatory genes were generally more induced in WT than SLFN11-KO cells, as illustrated for some of the genes of the IL-18 and, in addition, some genes of the IFNG and NF-κB signaling pathway (Supplemental Figure 6, D and E). These transcripts include IFN regulatory factor 1 (IRF1), a master regulator of inflammation necessary for the development of immune-mediated cancer rejection (36, 43), and CCL5 and CXCL10, which, by binding to CCR5 and CXCR3, respectively, are the main mediators of intratumor chemoattraction of activated CXCR3^+^CCR5^+^ cytotoxic T cells (44, 45). Such genes are almost invariably included in prognostic and predictive immune signatures (35, 46, 47). Furthermore, high-content microscopy imaging revealed an increase in nuclear accumulation of phosphorylated NF-κB (p–NF-κB) in WT cells (Supplemental Figure 6F), which, together with IFN, induces CCL5 (48) and CXCL10 (49) secretion. Taken together, these results demonstrate that DDA treatment triggers immune-related gene expression and an activation of an immune response in SLFN11-proficient cancer cells.

**Figure 6:**
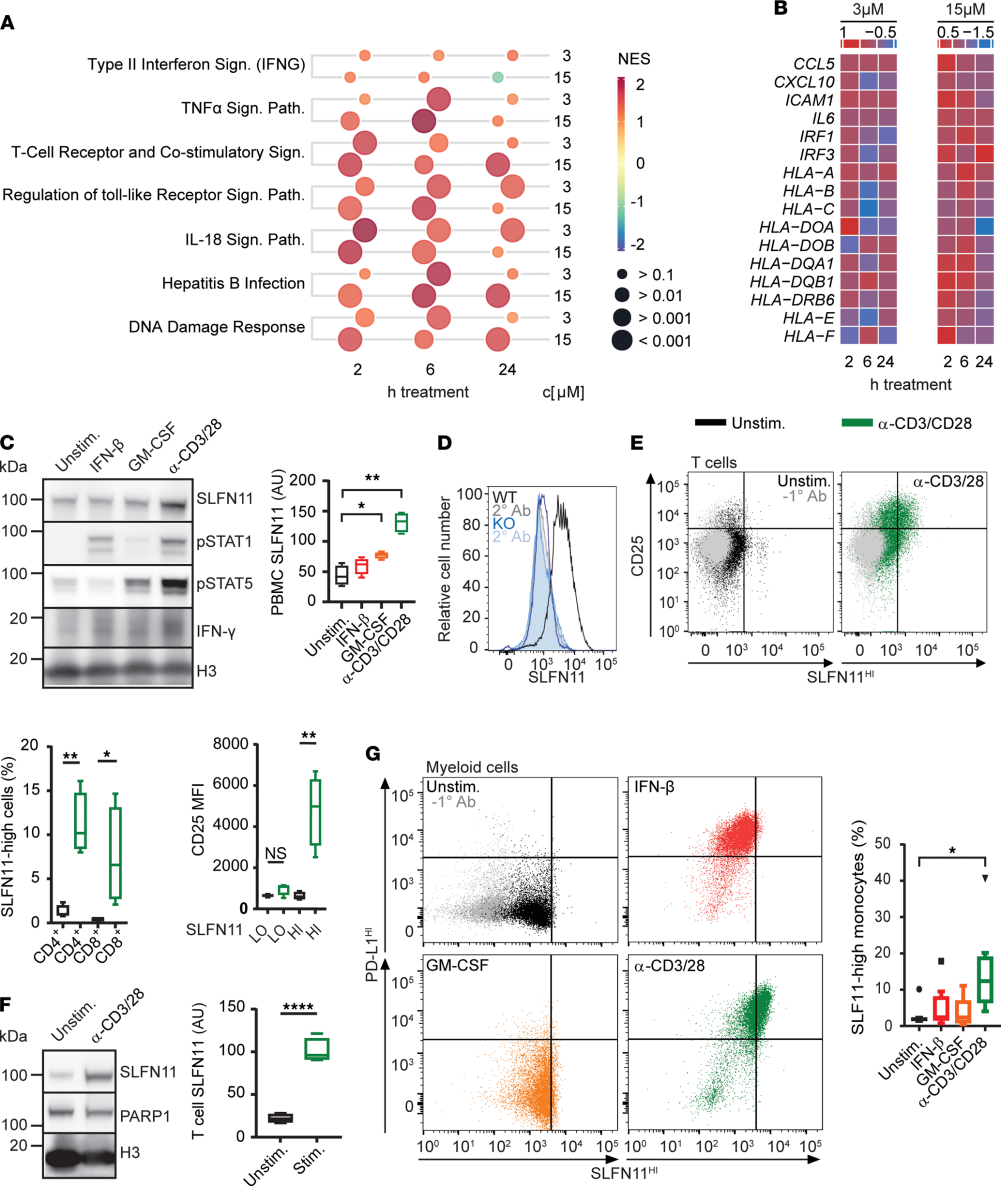
SLFN11 is a dual biomarker capturing simultaneously interconnected immunological and cancer cell–intrinsic functional dispositions associated with sensitivity to platinum treatment. (**A**) Bubble chart representing significantly enriched pathways during treatment with cisplatin in SLFN11^hi^ (*n* = 3) versus SLFN11^lo^ (*n* = 4) ovarian cancer cell lines. The bubble size indicates the *q* value (FDR), whereas the color represents the direction of the change as normalized enrichment score (NES), with red indicating a positive enrichment of the comparison and blue a negative one. c, concentration. (**B**) Heatmap of log fold changes of selected immune-related transcripts for the comparison between the SLFN11^hi^ and SLFN11^lo^ cancer cell lines at the same time points as for **A**. (**C**) PBMCs were incubated with IFN-β, GM-CSF, or anti-CD3/CD28 beads for 24 hours and immunoblotted as indicated. Chart shows SLFN11 densitometry (*n* = 4). (**D**) DU145 parental or SLFN11-KO cells were stained with/without anti-SLFN11 and analyzed by flow cytometry. (**E**) PBMCs were treated as in **C** and analyzed by flow cytometry. FACS plots show SLFN11 and CD25 expression in naive (top) and anti-CD3/CD28-stimulated (bottom) cultures. Charts show quantification of SLFN11^hi^ CD4^+^ and CD8^+^ T cells (top) and CD25 expression in SLFN11^hi^/SLFN11^lo^ T cells (bottom). (**F**) Purified CD3^+^ T cells were stimulated with anti-CD3/CD28 beads and immunoblotted as indicated. Chart shows SLFN11 densitometry (*n* = 4). (**G**) PBMCs were treated as in **C** and analyzed by flow cytometry for SLFN11 and programed cell death ligand 1 (PD-L1) expression in myeloid cells. Chart shows quantification of SLFN11^hi^ myeloid cells (*n* = 8). The box plots depict the minimum and maximum values (whiskers), the upper and lower quartiles, and the median. The length of the box represents the interquartile range. All data in graphs are presented as mean ± SEM. Statistics by Student’s *t* test, pairwise comparisons with the 2-tailed *t* test with reported *P* values adjusted for family-wise error using the Holm’s method. *P < 0.05; **P < 0.01; ****P < 0.0001.

We next sought to validate these results in ovarian cancer cell lines treated with platinum (cisplatin). We first selected 3 SLFN11-deficient (EFO-21, KURAMOCHI, RMGI, SLFN11^–^) and 3 SLFN11-proficient (OAW42, OV7 and OV-56, SLFN11^+^) ovarian cancer cell lines that were all STING pathway proficient, as illustrated by cGAS and IRF3 expression (Supplemental Figure 7A). As expected, SLFN11-proficient cell lines were more responsive (Supplemental Figure 7B) and more prone to undergoing apoptosis (Supplemental Figure 7C) following cisplatin treatment compared with the SLFN11-deficient cell lines. We then focused on OV7 cells that express SLFN11 at similar levels compared with DU145 prostate cancer cells (Supplemental Figure 7A). Transient downregulation of SLFN11 in OV7 did not affect cGAS or IRF3 expression (Supplemental Figure 8A) and led to more resistance (Supplemental Figure 8B) and less cytotoxicity upon cisplatin treatment (Supplemental Figure 8C). Cisplatin treatment induced *IL6* and *IL8* gene expression in OV7 cells in an SLFN11-dependent manner (Supplemental Figure 8D). Of note, *IL6* and *IL8* gene expression peaked at early time point treatment and then steadily decreased over the 48-hour time period, fitting with the observations already reported (11). NF-κB phosphorylation was elevated in SLFN11-proficient OV7 cells when compared with SLFN11-deficient OV7 SLFN11 knockdown (KD) (validation of the KD in independent experiments, see Supplemental Figure 8E) and EFO-21 cells (Supplemental Figure 8F). Finally, these results were reproduced in the NCI-60 panel of ovarian cancer cell lines (50), where SLFN11^hi^/proficient cells also showed an enrichment of immune-related pathways following cisplatin treatment with similar transcriptomic kinetic profiles as OV7 cells (Figure 6A). This observation is illustrated in more detail for selected genes of the IFNG signaling pathway in Figure 6B. Overall, these results indicate an SLFN11 dependency of the immune modulation induced by cisplatin in ovarian cancer cell lines.

Given the correlation of SLFN11 expression in the immune compartment of ovarian tumors with response to cisplatin, we investigated whether this finding could be representative of tumor-immune transactivation. Previous studies using ovarian tumor–bearing mice show cisplatin can activate the cGAS/STING pathway and T cell recruitment, indicative of innate priming and an adaptive immune response to this treatment (51). Moreover, the cGAS/STING/IRF3 target, IFN-β, has also been shown to induce SLFN11 expression in PBMC cultures (14), although it was not determined which immune cell subtype(s) upregulated this factor. We therefore examined the response of PBMCs to IFN-β versus T cell activation (anti-CD3/CD28 beads) and myeloid stimulation (GM-CSF) — 2 major subpopulations of PBMCs that influence the magnitude of an antitumor immune response (Figure 6, C–G). By Western blot, we found that PBMCs expressed SLFN11 but showed robust induction of this factor only under T cell activation conditions (Figure 6C). We next used flow cytometry to understand SLFN11 expression across PBMC subtypes and demonstrated exceptional specificity of SLFN11 detection in this system (Figure 6D). T cells were gated into CD3^+^CD4^+^ or CD3^+^CD8^+^ populations and stained for SLFN11 and the activation marker, CD25 (Figure 6E). Naive T cells (shown in black) expressed a low level of SLFN11, which was significantly increased (*P* = 0.002) by treatment with anti-CD3/CD28 beads (shown in green) but did not change with IFN-β or GM-CSF, as expected (data not shown). Quantification of CD25 expression in SLFN11^lo^ and SLFN11^hi^ populations revealed that it is only in activated T cells that SLFN11 is induced. Additionally, we confirmed SLFN11 induction by CD3/CD28 engagement in purified CD3^+^ T cell cultures by Western blotting (Figure 6F). Under these conditions, SLFN11 expression increased 5-fold in activated versus naive controls. Thus, by both flow cytometry and Western blotting, we showed that naive T cells express SLFN11, which is robustly induced by activation that mimics antigen presentation.

SLFN11 expression in myeloid cells was determined in CD14^+^CD86^+^ PBMCs (Figure 6G). Again, we found that neither IFN-β (red) nor GM-CSF (orange) robustly modulated SLFN11 in comparison with untreated cultures (black). However, in coculture with activated T cells (green), the frequency of SLFN11^hi^ myeloid cells was increased 4-fold (bar chart). Under these conditions, all SLFN11^hi^ myeloid cells were also positive for PD-L1 (Figure 6F, right), a canonical IFN-γ–inducible gene, suggesting that paracrine factors — such as IFN-γ, secreted by activated T cells in high quantity — may coordinately regulate the expression of both PD-L1 and SLFN11 in these cells (52).

Finally, we wondered whether, and to what degree, the SLFN11 expression pattern observed in PBMCs is mirrored in tumor-infiltrating leukocytes in the context of different functional orientation. To address this point, we correlated SLFN11 expression with markers of immune exhaustion and costimulatory and inhibitory checkpoints. We also displayed its correlation with myeloid-derived suppressor cells (MDSCs; see Methods) in TCGA ovarian cancer patient data set (Supplemental Figure 9). As expected, we found that markers of activation correlated strongly with markers of inhibition and exhaustion, both at single-gene and at cellular levels. This reflects the nature of the intratumor immune response, as counterregulatory signaling pathways are engaged following immune activation. SLFN11 positively correlated with almost all markers. In particular, SLFN11 was positively correlated with different MDSC estimates, especially with a signature of monocyte-derived MDSCs (MDSC_INT). This is in line with the high expression of SLFN11 observed in peripheral blood monocytes. The correlations with SLFN11 were in general weaker than the ones observed among markers of inhibition, activation, and exhaustion, most likely due to the complex regulation and expression of SLFN11 in both cancer cells and immune cells.

Taken together, our results indicate that SLFN11 is a dual biomarker capturing simultaneously interconnected immunological and cancer cell–intrinsic functional dispositions associated with sensitivity to DDAs.

## Discussion

Since its discovery in 2009 (53), SLFN11 has been reported to play a role in the native immune response (14, 16), as well as a potential role in adaptive immunity in cancer (6, 13). In addition, work from various groups confirmed SLFN11 as being a determinant of sensitivity to a broad range of DDAs with different modes of action (1–6, 54), as well as PARP inhibitors (6–9), in different, mainly preclinical, cancer settings. An understanding how SLFN11 modulates the response to CT in patients is of paramount importance for both basic biology and clinical viewpoints and is currently missing.

The assessment of SLFN11 as a clinical biomarker is hindered by the lack of validated algorithms to score it in human tissues. By implementing analytic pipelines in clinical material, we demonstrate, for the first time to our knowledge, that SLFN11 in both the neoplastic and microenvironmental compartments of tumor specimens modulates the response to platinum-containing regimens in patients with HGSOC. Recent reports highlight SLFN11 expression in B cells, T cells, and macrophages in serial sections of human tissues (55), but here, for the first time to our knowledge, we developed a multiplex IHC assay and demonstrated concomitant expression of SLFN11 in CD3^+^CD8^+^ T cells, CD20^+^ B cells, and CD68^+^ monocytes/macrophages infiltrating HGSOC specimens. Our results indicate high SLFN11 expression in macrophages and monocytes, in line with a previous report (16), whereas low expression was observed in neutrophils. Of note, we also observed SLFN11 presence in other stromal cell types that remain to be identified. SLFN11 has been shown to predict response to DDA in different cancer models (1, 3–5, 54, 56), but to our knowledge, we describe here for the first time that SLFN11 has more predictive power when its expression in the stromal noncancer compartment is taken into account. This might be especially relevant in immunologically “hot” tumors with high immune infiltration. Our results support overall SLFN11 profiling in clinical tissues, and we provide evidence that total levels of SLFN11 can be quantified by either transcript or protein analyses. Also, our findings point out that SLFN11, which is only expressed in humans and some primates, should be best assessed in clinical tissues, rather than xenograft or in vitro cancer tissues, to understand its relevance in a clinical setting. In our analyses, we demonstrate that SLFN11 in cancer and immune cells is independently a predictor of response to CT in HGSOC. These observations are in agreement with recent findings. Accordingly, in 2 other studies SLFN11 has been shown to be a biomarker of longer survival in CT-treated ovarian cancer patient cohorts (1, 56). The concept that the immune-infiltrating milieu can modulate cancer prognosis is not new: multiple studies have reported on the role of TILs in the prognosis of ovarian cancer and HGSOC (21–24), and Liu et al. expanded this notion by looking at other leukocyte subpopulations (57). In analogy with these studies, we found that SLFN11 and TILs were associated with a better prognosis in our HGSOC cohorts. Similar trends were noted for naive B cells and macrophages of type 1; on the contrary, macrophages of type 2 were associated with poorer prognosis. We also noted that SLFN11 was correlated with well-established biologically selected immune signatures (Figure 5B), indicating that SLFN11 is associated with immune activation in HGSOC.

We investigated the mechanism of SLFN11 association with immune activation. By performing transcriptomic and single-cell analyses, we found that gemcitabine treatment in prostate cancer cells induced the expression of immune-related pathways in SLFN11-proficient cancer cells, which is in line with recent findings made with other compounds inducing replication stress (11). However, given that our results were translatable to cisplatin treatment in ovarian cancer lineages, we propose that general DDA treatment may trigger this phenotype in cancer cells, which may directly contribute to tumor-immune transactivation induced, e.g., by cisplatin treatment (51).

The cGAS/STING/IRF3 target, IFN-β, which is induced following cisplatin treatment (51), has been shown to induce SLFN11 protein expression in PBMCs. Herein, we investigated the response of PBMCs to IFN-β versus T cell and myeloid stimulation. We found that naive myeloid cells and T cells express SLFN11, which is robustly induced in both cell types by activation that mimics antigen-driven T cell receptor engagement. Neither IFN-β nor GM-CSF recapitulated the magnitude of this increase in SLFN11 expression. We further propose that SLFN11 expression in tumor-infiltrating immune cells mirrors the cellular subset distribution in PBMCs (Supplemental Figure 9). Our data suggest that T cell activation may represent a primary mechanism for SLFN11 upregulation in both adaptive and innate immune cells by cell-autonomous and non–cell-autonomous pathways, respectively. Indeed, as SLFN11 induction correlates with PD-L1 expression in myeloid cells cocultured with activated T cells, paracrine factors such as IFN-γ may coordinately regulate this response (52). Recent findings demonstrate that patients with a high dendritic cell gene signature have increased overall survival following checkpoint inhibitor (PD-L1 blockade) treatment (58). As dendritic cells are required for efficient antigen cross-presentation and T cell activation, the correlation between SLFN11 and PD-L1 in our analyses suggests that SLFN11 may therefore also be a proxy indicator of response to PD-L1 therapy.

To the best of our knowledge, there are currently no biomarkers of prognosis in HGSOC, apart from stage and optimal debulking status. Our findings indicate that SLFN11 emerges at a crossroads between CT and immune response and its relevance is multilayered. On one hand, its absence is informative of lack of response to the backbone of CT in HGSOC: platinum agents. On the other hand, its expression may help guide the use of immune checkpoint inhibitors, which have shown promising anticancer activity in HGSOC but whose optimal therapeutic setting is still under debate, due to the failure of “relevant biomarkers” to closely predict their activity (19).

We propose that the coordinated regulation of SLFN11 across tumor cells and the activated adaptive immune system highlights the potential of this factor as a biomarker for both tumor activity and immune transactivation to platinum-based therapy.

Of note, SLFN11 was identified as a sensitizer of tumor cells to T cell– and IFN-γ–mediated cytotoxicity (12). Accordingly, following IFN-γ exposure, SLFN11 has been shown to couple IFN-γ receptor signaling to the induction of DNA damage and cell death in tumor cells in a context-dependent fashion. Taken together, these results point at a complex interplay between SLFN11 in cancer cells and the immune system in cancer, which fosters further investigation.

We are aware of the limitations of our study. Among them, the retrospective nature of the analysis is unavoidable. The second limitation is the small sample size of our clinical cohort. Nevertheless, the fact that our observations are translated to a larger HGSOC cohort from TCGA is reassuring. Also, to our knowledge, we showed here for the first time, through an in silico effort and by direct measurement, the presence and localization of SLFN11 in tumor and tumor-infiltrating milieu in patients with HGSOC. Moreover, we provided mechanistic insights into the link between SLFN11 cancer immunity and DNA-damaging treatment.

In summary, the current study shows that SLFN11 in both cancer cells and a multitude of immune cells and potentially other (to-be-defined) cell types are associated with a better prognosis of HGSOC patients treated with platinum-containing regimens. Our findings add important information on the action of SLFN11 beyond its recently described role; hence, we propose SLFN11 as a dual biomarker capturing simultaneously interconnected immunological and cancer cell–intrinsic functional dispositions associated with sensitivity to DDAs.

## Methods

### HGSOC specimens.

Patients receiving a diagnosis of HGSOC, treated with neoadjuvant platinum-based CT at Léon Bérard Cancer Center, Lyon, France, from January 2008 to June 2014, and meeting the following criteria, were retrospectively included, in a consecutive fashion, for the reported analyses: written informed consent for biobanking and use of samples for research purposes according to the hosting institution, histologically confirmed HGSOC (grade 3 according to the American Joint Committee on Cancer Tumor-Node-Metastasis stage) (59), radiological and/or surgical classification as stage IIIc/IVa (Fédération Internationale de Gynécologie et d’Obstétrique classification) (60) at diagnosis, Eastern Cooperative Group performance status 0–1 at diagnosis, postmenopausal status at diagnosis, platinum-based neoadjuvant treatment at diagnosis followed by surgery, availability of a FFPE prechemotherapy tumor block from diagnostic biopsy, and availability of clinical information concerning treatment and response duration and disease status at the time of sample collection. Cases or patients were excluded if they had primary debulking surgery followed by CT, if surgical debulking was suboptimal — i.e., leaving more than 1 cm of residual tumor at any site according to the American Cancer Society — if they were stage IVb, if they had received a previous or concomitant diagnosis of neoplasia (with the exclusion of carcinoma in situ of the cervix or skin basalioma), or if they had received previous CTs for any reason. Patients were divided into PS and PR groups as previously described (61, 62). In particular, according to the most commonly used definition, patients were defined as PR if progressing/recurring within 6 months from the end of the first platinum-based CT.

### Cell culture and compounds.

DU145 cells were cultured in Eagle’s MEM (ATCC) supplemented with 10% FBS. In the ovarian cancer cell line panel, EFO-21 and KURAMOCHI cells were cultured in RPMI 1640 media (Corning) supplemented with 10% FBS for KURAMOCHI cells and for EFO-21 cells with 20% FBS, 2 mM glutamine, 1x MEM nonessential amino acids, and 1 mM sodium pyruvate. RMGI cells were grown in Ham’s F12 media (MilliporeSigma) containing 10% FBS and 1 mM glutamine and OAW42 cells in DMEM (Gibco, Thermo Fisher Scientific) supplemented with 10% FBS, 2 mM glutamine, 1 mM sodium pyruvate, and 20 U/L insulin. OV56 and OV7 cells were cultured in DMEM/F-12 1:1 media (Gibco, Thermo Fisher Scientific), 5% FBS, 2 mM glutamine, 0.5 μg/mL hydrocortisone, and 10 μg/mL insulin. The assay media for EFO-21, KURAMOCHI, RMGI, and DU145 cells was RPMI 1640 media and for OAW42, OV56, and OV7 cells DMEM, both supplemented with 10% FBS and 2 mM glutamine. Hydrocortisone, FBS, and insulin were from MilliporeSigma and glutamine, sodium pyruvate, and MEM nonessential amino acid solution from Gibco, Thermo Fisher Scientific. DU145 and EFO-21 cell lines were purchased from DSMZ, KURAMOCHI and RMGI cell lines from JCRB, and OAW42, OV56, and OV7 cell lines from ECACC. All cell lines were authenticated by short tandem repeat DNA fingerprinting analysis and validated to be free of Mycoplasma and viruses. Gemcitabine and cisplatin were obtained from Tocris. Stock solutions of gemcitabine were prepared in aqueous solution (50 mM) and DMSO (10 mM) and for cisplatin in aqueous solution (1.67 mM). KO of SLFN11 in DU145 cells was performed by CRISPR/Cas9 in-house and transient KD of SLFN11 by siRNA transfections using RNAi-Max kit (Thermo Fisher Scientific, TF) as previously described (1).

### Ex vivo/in vitro tissue culture and FACS analysis.

Human PBMCs were enriched by density centrifugation (Lymphoprep, STEMCELL Technologies, ST) from leukocyte cones (supplied by NHS Blood and Transplant Service, United Kingdom, as anonymized samples from consenting donors). CD3^+^ T cells were isolated by negative selection (ST-17951). All cells were cultured in RPMI (MilliporeSigma R8758) supplemented with 10% FBS and incubated at 37°C with humidification and 5% CO_2_. Cultures were left untreated or incubated with IFN-β (100 ng/mL, R&D Systems 8499-IF-010), anti-CD3/CD28 Dyna beads (TF 11131D) or GM-CSF (100 ng/mL, ST, 78015.1).

For flow cytometry, PBMCs were plated into 96-well, round-bottomed, polypropylene plates (Costar 3879). After 24-hour treatments, cells were washed in enzyme-free dissociation buffer (TF 13151014) and stained for viability (TF L34976), blocked (FcX, BioLegend [B] 422302), and immunostained for surface markers (CD3, B 317306; CD8, B 344706; CD4, B 317428; CD25, B 302610; CD14, B 367114; CD206, B 321104; CD86, B 374208; CD274/PD-L1, B 393608). Cells were fixed and permeabilized (eBioscience, TF, 00-5523-00) and stained for intracellular SLFN11 (Cell Signaling Technology 34858) and anti–rabbit IgG (B 406410). Flow data were captured on a BD FACSCelesta and analyzed in FlowJo. T cells were gated by CD3^+^ and CD4^+^/CD8^+^ expression and myeloid cells by CD14^+^ expression. Gating for SLFN11 upregulation (SLFN11^hi^) was determined by a threshold containing more than 95% of control cells.

### CellTiter-Glo viability and Incucyte cell confluence and apoptosis/cell death assay.

For CellTiter-Glo luminescence assays (Promega), cells in 96-well plates were manually dosed or dosed using a D3000e Digital dispenser (HP Life Science Dispensing), and cell viability was determined 72–96 hours later. Percentage growth was determined using the equation (T – T0)/(C – T0) × 100, where T = compound-treaded cells; T0 = cells at 0-hour time point, and C = control cells. Incucyte cell confluence, annexin V green apoptosis, and Cytotox red (Essen BioScience, now Sartorius) assays were performed according to the manufacturer’s instructions. Cell growth and relative apoptosis/cell death were calculated as previously described (63).

### Immunoblotting and immunofluorescence.

If not otherwise indicated, whole-cell extracts were prepared as described (39). For PBMC immunoblotting (IB), purified CD3^+^ T cells were incubated with anti-CD3/CD28 Dyna beads at ratio of 1:1 (TF 11131D) for 4 days, prior to lysis in RIPA buffer (TF 89901; plus complete protease and phosphatase inhibitors, MilliporeSigma). For unstimulated controls, T cells were lysed at day 0. Cell lysates were analyzed by standard SDS-PAGE IB. Immunostaining was visualized by HRP-labeled secondary antibodies (Jackson ImmunoResearch, 7076 and 7074) and enhanced chemiluminescence (TF) using Syngene GBOX and quantified by densitometry (ImageJ, NIH).

For immunofluorescence (IF), cells were fixed and permeabilized with 4% paraformaldehyde in PBS (Affymetrix) and 0.35% Triton X-100 (MilliporeSigma), blocked in 3% BSA-PBS, and incubated overnight with primary antibodies, followed by secondary Alexa Fluor 488– and Alexa Fluor 594–conjugated antibodies (Molecular Probes, TF, A32731 and A-21125) for 1 hour and counterstaining with DAPI. Images were acquired with a CellInsight High-Content Screening Platform (TF) using the 10x objective. Image analysis was performed on a Columbus image data storage and analysis system (PerkinElmer) using optimized image algorithms to identify the response to DNA damage and immune oncology markers. The used antibodies were: anti-SLFN11 (Abcam, ab121731; and Novus, NBP2-57084 1/1000 for IB), anti-cGAS (Cell Signaling Technology [CST], 15102 1/1000 for IB), anti-IRF3 (CST, 11904 1/1000 for IB), anti-vinculin (MilliporeSigma, V9131 1/2000 for IB), anti–p-NF-κB p65 Ser536 (CST, 3033 1/250 for IF), anti-PARP1 (CST, 9532 1/1000 for IB), anti–p-STAT1 Tyr701 (CST, 9167 1/1000 for IB), anti–p-STAT5 (CST, 9359 1/1000 for IB), anti–IFN-γ (CST, 8455 1/1000 for IB), and anti-histone H3 (CST, 3638 1/2000 for IB).

### RNA sequencing and RT-PCR.

For RNA sequencing, DU145 WT and 2 different SLFN11-KO clones were treated for 0, 6, or 24 hours with 100 nM gemcitabine (3 independent experiments) before the cells were harvested for total RNA isolation and subjected to RNA-sequencing analysis. Total RNA was isolated from cells using the RNeasy Mini Kit from QIAGEN. Library preparation was done using the Illumina TruSeq mRNA Stranded HS kit, and sequencing was performed on 4 lanes of HiSeq4000, SE50. Data were analyzed by ROSALIND (https://rosalind.onramp.bio/), with a HyperScale architecture developed by OnRamp BioInformatics, Inc.

RNA preparation and RT-qPCR on cells were performed as previously described (64). Briefly, total RNA was prepared from cells grown in 96-well plates with FastLane Cell Probe Multiplex kit (QIAGEN). Dual-color RT-qPCR reactions were performed on a Lightcycler 480 instrument (Roche). Gene expression values were calculated using the –ΔΔCt method, using housekeeping gene Ct values and control-treated Ct for normalization. The following MGB assay probes from TF Applied Biosystems were used: *CXCL8* (HS99999034_m1), *IL6* (HS00174131_m1), and *18S rRNA* (HS99999901_s1, housekeeping gene). RT-qPCR on FFPE tissues was performed as described in the Supplemental Methods.

### IHC and image analysis for SLFN11, CD3, CD8, CD20, and CD68.

SLFN11 IHC was assessed in all cases from a pre-CT specimen, obtained either through diagnostic biopsy or at pre-CT debulking surgery. IHC was performed on 4 μm thick sections of FFPE tissues and carried out on BOND RX (Leica Biosystems) using ER1 (pH 6, Leica) antigen retrieval. Slides were stained with primary rabbit polyclonal anti-SLFN11 antibody (Abcam ab121731) at 2.5 μg/mL. Detection was performed with anti-rabbit poly-HRP-IGG, DAB refine and DAB enhancer (Leica, polymer refine detection kit). Digital slide images were acquired with the Aperio AT2 scanner (Leica) using a 20x objective. A HALO (Indica Labs) cytonuclear image analysis algorithm was optimized and run alongside different tissue classifiers and annotations, to capture the percentage of cancer, noncancer, and overall (cancer + noncancer) nuclei with strong (3+), moderate (2+), weak (1+), or negative staining to calculate SLFN11 H-scores as [(%1+ cells) + (%2+ cells × 2) + (%3+ cells × 3)] in each sample. Samples were H&E stained to identify cancer cells. The same algorithm was used across all specimens, and the analysis was blindly performed. All samples were in addition manually evaluated by a pathologist for cancer SLFN11 H-scores. CD3 and CD8 IHC was performed on full-thickness sections of FFPE tissues and carried out on Ventana Benchmark Ultra (Ventana Medical Systems) using heat-based antigen retrieval. Slides were stained with primary rabbit monoclonal anti-CD3 antibody (clone 2GV6 at 2.5 μg/mL) and primary rabbit monoclonal anti-CD8 antibody (clone SP57 at 2.0 μg/mL), both from Ventana Medical Systems. Slides were evaluated by a pathologist for total and intratumor CD3 and CD8 by calculating mean CD3 and CD8 values from 3 high-power field regions per sample. To confirm spatial resolution of SLFN11 protein in immune-infiltrating cells, 4 μm serial FFPE sections of human tonsil tissue or HGSOC specimens were taken. Sections were IHC stained for CD3, isotope control, SLFN11, CD8, CD20, and CD68 with BOND RX using ER1 (Leica Biosystems; CD8 and CD20) or ER2 (CD3, IGG, SLFN11 and CD68) antigen retrieval. Primary antibodies used were as follows: anti-SLFN11 as described above, anti-isotype control (ab172730, Abcam, at 2.5 μg/mL), anti-CD3 (clone 2GV6, Roche, at 0.1 μg/mL), anti-CD8 (clone C8/144 B, Dako, at 0.75 μg/mL), anti-CD20 (clone L26, Abcam, at 0.1 μg/mL), and anti-CD68 antibodies (clone PG-M1, Dako, at 0.3 μg/mL). Detection was performed with poly-HRP-IGG, DAB refine and DAB enhancer (polymer refine detection kit, Leica). Digital slide images were acquired with the Aperio AT2 scanner (Leica) using a 20x or 40x objective.

### Multiplex IHC staining and multispectral image acquisition.

Multiplex IHC staining was conducted on 3 μm thick sections from FFPE HGSOC tissue using the Opal Polaris 7-color IHC Detection Kit (PerkinElmer). The BOND RX automated stainer was used for the pretreatment and the staining of the tissue using ER2 (pH 9, Leica Biosystems) antigen retrieval. The endogenous peroxidase was blocked using the Peroxidase Block Novocastra (Leica), before staining the tissue through repeated staining cycles for each marker. Each staining step cycle was composed of 5 steps: protein blocking using the Antibody Diluent/Block reagent (PerkinElmer), primary antibody incubation, secondary antibody incubation, Opal dye incubation, and an antibody denaturation step using ER1 (pH 6, Leica). The primary antibodies used for the chromogenic IHC staining were also used for the multiplex IHC, except when indicated, and in the following order: anti-CD3 (at 0.1 μg/mL) visualized with Opal480 (1/100), anti-CD8 (at 1.5 μg/mL) visualized with Opal570 (1/100), anti-CD68 (at 0.3 μg/mL) visualized with Opal520 (1/200), anti-SLFN11 (CST 34858, at 1/100 dilution) visualized with Opal620 (1/100), and anti-CD20 (at 0.1 μg/mL) visualized with Opal690 (1/100). The Opal polymer HRP secondary antibody (PerkinElmer) was used for the CD20 staining, the anti-rabbit HRP SignalStain Boost IHC Detection Reagent (CST) for the CD3 and SLFN11 stainings, and the anti-mouse HRP SignalStain Boost IHC Detection Reagent (CST) for CD8 and CD68 stainings. At the end of the protocol, the stained slide was counterstained with DAPI. The slide was scanned using the Vectra Polaris automated imaging system (Polaris 1.0.11; PerkinElmer), and multispectral images were unmixed using the InForm software version 2.4.8 and the synthetic spectral library (PerkinElmer).

### Gene expression analyses and RNA-sequencing profiling of the NCI-60 cancer cell lines panel and human leukocyte subpopulations.

For gene expression analysis of cisplatin-treated ovarian cancer cell lines from the NCI-60 panel, the CEL files were downloaded from GEO (accession number GSE116439). Background preprocessing of raw data using probe sequence information was performed with the gcrma package (65), while normalization and probe set summarization were obtained with the frozen robust multiarray analysis method (66). To assess differentially expressed genes (DEGs) between SLFN11^hi^ and SLFN11^lo^ ovarian cancer cell lines at various time points with 2 concentrations of cisplatin (3 μM and 15 μM), values were fitted with a generalized linear model with a blocked factorial design, taking into account intersample correlation of cell lines as a random effect within the environments of the *limma* package (67) and microarray studies (68). Subsequently, DEGs at various time points were ranked by log fold change and analyzed for pathway enrichment analysis using the *clusterProfiler* package with the fast gene set enrichment analysis algorithm described (69–71).

RNA-sequencing results for SLFN11 in sorted leukocyte subpopulations from patients with immune-associated diseases, as further described by Linsley et al. (72), were obtained from the GEO (accession number GSE60424).

### Data availability.

Gene expression data are available in the GEO (GSE179896).

### Statistics.

Statistical analyses and power considerations are described in the Supplemental Methods.

### Study approval.

The presented research was conducted according to the ethical considerations and in compliance with the principles of the Declaration of Helsinki and approved by Regione Liguria Ethics Committee, Genova, Italy, with registration number 347/2018 (approved 19/06/2019).

## Author contributions

CW, DB, and GZ conceived and designed the study; CW, MK, JB, DF, AG, FG, JRC, LF, OD, DB, and GZ designed methodology and software and collected data; CW, DB, EL, and GZ wrote the original draft and performed visualization; CW, MK, JB, DF, AG, FG, JRC, LF, NC, IRC, OD, DB, AB, EL, and GZ reviewed and edited the draft; NC, IRC, EL, and GZ performed project administration; and EL and GZ acquired funding.

## Supplementary Material

Supplemental data

Supplemental tables 1-7

## Version 1 09/22/2021

Electronic publication
