# Supplementary material for: SLFN11 captures cancer-immunity interactions associated with platinum sensitivity in high-grade serous ovarian cancer
Source: JCI Insight. 2021 Sep 22;6(18):e146098. doi: 10.1172/jci.insight.146098 (PMC8492341; doi:10.1172/jci.insight.146098)
Supplement: Supplemental data [file jciinsight-6-146098-s029.pdf]

Winkler et al. Supplementary material and methods, figures and legends:

Supplementary material and methods

RT-PCR on FFPE tissues. RT-qPCR on on FFPE tissues was performed as previously described (1). In brief, three membrane glass slides (PEN Membrane Glass slides, Arcturus® Bioscience Inc. CA, USA) were loaded with 8-µm thick sections cut from FFPE-embedded pre-treatment diagnostic biopsies. One section of each sample was hematoxylin/eosin-stained and examined to assess tumor cellularity. Samples with a < 70% cellularity were subjected to microdissection. Upon RNA extraction and retro-transcription, RNA was quantified by Q-RT-PCR using a SLFN11-specific Invitrogen™ TaqMan® assay (Invitrogen Inc. CA, USA) on an Applied Biosystems Inc. HT-7900 instrument. Samples were analyzed in triplicate, using RPLP0, GAPDH and GUS as housekeeping (HK) genes. The mean PCR cycle thresholds (Ct) of the three HK genes was subtracted from the Ct value of SLFN11 for each sample, expressed as $\log_2$ (ΔCt) and, in turn, the median of ΔCts from the dataset was subtracted from ΔCts of single samples and inverted, to obtain a normally distributed, zero-centered semi-quantitative value for each sample (-ΔΔCt), as previously described (2).

Statistical analyses. Correlations between continuous variables were calculated using the Spearman's rank coefficient and represented using scatter plots (package CNtu (3)), whereas differences in continuous distributions were calculated using the Wilcoxon test without continuity correction. Intraclass correlation coefficients (ICCs) were calculated to assess the consistency and agreement of IHC assessments (package psy (4)), and visually inspected for bias and trend using dot plots and Bland-Altman plots. Correlation matrices and correlograms of IHC and TILs were generated using the package corrgram (5). P-values were adjusted for multiple testing using the Benjamini-Hochberg method. Univariable Cox's proportional hazards regression models were used for associations with PFI, after log2 transformation and scaling of continuous measures. HR, 95%CI and p-values according to the Wald statistics were reported. For multivariable Cox's regression, variables with a p-value < 0.1 were entered in a stepwise forward-backward model minimizing the Akaike Information Criterion (package MASS (6)). "Optimal" (quoted because considerable as such only in the examined case set) cutoffs to dichotomize continuous variables were obtained using binary class labels (i.e. NR vs PR patients) and maximizing the accuracy to

correctly classify those classes with the package cutpointr (7). Forest plots representing adjusted HR and 95%CIs were generated with the package survminer (8). To estimate the relative abundances of cell types in gene expression mixtures from the Cancer Genome Atlas high-grade ovarian cancer data (OVCAR) - processed and normalized as described in Roelands J, et al., (9) the count matrix was analyzed using CIBERSORTx (10) on the dedicated web tool available at the URL https://cibersortx.stanford.edu/. The following parameters were set for the analysis: impute cell fractions, signature file LM22.update-gene-symbols.txt, batch correction enabled, batch correction mode B-mode, disable quantile normalization true, run mode absolute, permutation number 1,000. The job was performed on March 6, 2020. To derive cancer cellularity, we used ESTIMATE (11) with default parameters, after log2 transformation and offsetting count data by a value = 1. Single sample gene set enrichment analysis for selected immune phenotypes, gene, and hallmark immune signatures was obtained as previously described (9, 12-14). For univariable analysis of correlations between SLFN11 expression and cell fractions, we calculated the Spearman's correlation coefficient. P-values were adjusted for multiple testing using the Benjamini-Hochberg method. For multivariate analysis of CIBERSORTx cell fractions and SLFN11 expression in the HGSOC TCGA dataset, we included variables with FDR < 0.05 for correlation with SLFN11, including cancer cellularity, after normalizing vectors as follows: we first removed near-zero variance variables (*caret* package (15)), then we pseudo-normalized data using the Tukey's ladder of power transformation method (*rcompanion* package (16)), finally, we centered and scaled them. The relationship between variables was represented using a variable correlation plot, with SLFN11 expression as a supplementary quantitative variable (17). To estimate the enrichment of myeloid-derived suppressive cells (MDSC) in ovarian cancer patients, gene expression deconvolution analyses were performed with single sample gene set enrichment analysis (ssGSEA) implemented in the "GSVA package" using cell-specific signatures. We used a specific signature (MDSC_INT) that was constructed based on 25 genes highly correlated with each other in cancer patient peripheral blood mononuclear cells, selected from the top 100 genes upregulated in extracellular vesicle (EV)-MDSCs vs monocytes(18, 19). Estimation of MDSC based on this signature was highly correlated with proportion of MDSC by flow cytometry in cancer patients. Additional MDSC signatures include the one proposed by Angelova *et al.*(20) (MDSC_Angel), based on markers selected

according to the literature, and a granulocytic myeloid-derived suppressor cell (G-MDSC) signature defined by comparing G-MDSC vs naïve neutrophils(21). Overall, these estimations cover both monocytic and granulocytic MDSC. For survival analyses, we selected TCGA OVCAR (HGSOC) cases with the following characteristics: stage IIIc/IV, histologic grade III, and with PFI > 28 days. SLFN11 was considered "high" when in the top two tertiles of expression, and "low" otherwise. Univariable Cox's regression and Kaplan-Meier curves were used as described above. For multivariable Cox's regression, we first transformed bimodal CIBERSORTx cell fractions into binary factors ("present" vs. "absent") if the Hartigan's dip test for unimodality was rejected with p-value < 0.01. We then performed feature selection starting from stage, age, SLFN11 transcript, and CIBERSORTx variables. To do so, we fitted the Cox's regression model by regularizing it with a lasso penalty, using the package glmnet (22) with default options (α = 1, 10-fold cross-validation), and iterating it 1,000 times to obtain the minimum average error of the regularization parameter lambda for variable selection. Finally, selected variables were entered in a Cox's multiple regression model to report HR point estimates and 95% CI. The dendrogram of similarity between immunologic signatures was built through hierarchical clustering using the Ward's criterion agglomeration method and Euclidean distance between variables (23). For comparisons shown in Figs 6C and 6G, adjusted p-values from pairwise t-tests corrected with the Holm's method to control for familywise error are reported.

Power considerations. The sample size for the present study was meant to identify a clinically significant difference in SLFN11 expression between platinum-resistant (PR) patients, defined as relapsing within six months from the end of chemotherapy, and platinum-sensitive (PS) ones (i.e., relapsing beyond six months from the end of treatment). The suggested size of the collected cohort was based on our previous findings of a very significant hazard ratio in terms of overall survival between "SLFN11-high" and "SLFN11-low" HGSOC patients, with SLFN11 levels deemed so if being above or below the median for the considered cohort (24). The required sample size would be of 24 patients, equally allocated in two groups of 12 PR and 12 PS ones, assuming a proportion of "SLFN11-high" patients of 10% in the first group and 70% in the second groups, with two-tailed $\alpha = .05$ and $1 - \beta = 0.9$ (z test family, G*Power 3.1.4). Assuming that 20% of samples could not be analyzed due to

failure in sample processing or testing, it was estimated that 28 samples were a sufficient number needed to test the aforementioned hypothesis.

References

1. Garutia A, Roccoa I, Cirmena G, et al. Quantitative Real Time PCR assessment of hormonal receptors and HER2 status on fine-needle aspiration pre-operatory specimens from a prospectively accrued cohort of women with suspect breast malignant lesions. *Gynecologic Oncology*. 2014;132(2):389-396.
2. Barretina J, Caponigro G, Stransky N, et al. The Cancer Cell Line Encyclopedia enables predictive modelling of anticancer drug sensitivity. *Nature*. Mar 28 2012;483(7391):603-7. doi:10.1038/nature11003
3. Desmedt C, Zoppoli G, Gundem G, et al. Genomic Characterization of Primary Invasive Lobular Breast Cancer. *J Clin Oncol*. Jun 1 2016;34(16):1872-81. doi:10.1200/JCO.2015.64.0334
4. Shrout PE, Fleiss JL. Intraclass correlations: uses in assessing rater reliability. *Psychological bulletin*. 1979;86(2):420-428.
5. Friendly M. Corrgrams. *The American Statistician*. 2002;56doi:10.1198/000313002533
6. Venables WN, Ripley BD. *Modern Applied Statistics with S, Fourth edition*. Springer, New York; 2002.
7. *Cutpointr: Determine and Evaluate Optimal Cutpoints in Binary Classification Tasks*. Version R package version 1.0.1. 2019. https://CRAN.R-project.org/package=cutpointr
8. *Survminer: Drawing Survival Curves using 'ggplot2'*. Version R package version 0.4.6. 2019. https://CRAN.R-project.org/package=survminer
9. Roelands J, Hendrickx W, Mall R, et al. Genomic landscape of tumor-host interactions with differential prognostic and predictive connotation. *bioRxiv doi:101101/546069*. 2019;
10. Newman AM, Steen CB, Liu CL, et al. Determining cell type abundance and expression from bulk tissues with digital cytometry. *Nat Biotechnol*. Jul 2019;37(7):773-782. doi:10.1038/s41587-019-0114-2
11. Yoshihara K, Shahmoradgoli M, Martinez E, et al. Inferring tumour purity and stromal and immune cell admixture from expression data. *Nat Commun*. 2013;4:2612. doi:10.1038/ncomms3612

12. Thorsson V, Gibbs DL, Brown SD, et al. The Immune Landscape of Cancer. *Immunity*. Apr 17 2018;48(4):812-830 e14. doi:10.1016/j.immuni.2018.03.023

13. Bindea G, Mlecnik B, Tosolini M, et al. Spatiotemporal dynamics of intratumoral immune cells reveal the immune landscape in human cancer. *Immunity*. Oct 17 2013;39(4):782-95. doi:10.1016/j.immuni.2013.10.003

14. Liberzon A, Birger C, Thorvaldsdottir H, Ghandi M, Mesirov JP, Tamayo P. The Molecular Signatures Database (MSigDB) hallmark gene set collection. *Cell Syst*. Dec 23 2015;1(6):417-425. doi:10.1016/j.cels.2015.12.004

15. *Package 'caret': Classification and Regression Training*. Version R-package version 6.0-86. 2020. https://github.com/topepo/caret/

16. *Package 'rcompanion': Functions to Support Extension Education Program Evaluation*. 2020. http://rcompanion.org/

17. Lê S, Josse J, Husson F. FactoMineR: An R Package for Multivariate Analysis. *Journal of Statistical Software*. 2008;25(1)

18. Huber V, Vallacchi V, Fleming V, et al. Tumor-derived microRNAs induce myeloid suppressor cells and predict immunotherapy resistance in melanoma. *J Clin Invest*. Dec 3 2018;128(12):5505-5516. doi:10.1172/JCI98060

19. Rinchai D, Verzoni E, Huber V, et al. Pazopanib induces dramatic but transient contraction of myeloid suppression 2 compartment in favor of adaptive immunity. *Clinical and Translational Medicine (in press)*. 2021;doi:10.1101/2020.05.01.071613

20. Angelova M, Charoentong P, Hackl H, et al. Characterization of the immunophenotypes and antigenomes of colorectal cancers reveals distinct tumor escape mechanisms and novel targets for immunotherapy. *Genome Biol*. Mar 31 2015;16:64. doi:10.1186/s13059-015-0620-6

21. Fridlender ZG, Sun J, Mishalian I, et al. Transcriptomic analysis comparing tumor-associated neutrophils with granulocytic myeloid-derived suppressor cells and normal neutrophils. *PLoS One*. 2012;7(2):e31524. doi:10.1371/journal.pone.0031524

22. Friedman J, Hastie T, Tibshirani R. Regularization Paths for Generalized Linear Models via Coordinate Descent. *Journal of Statistical Software*. 2010;33(1)

23. Murtagh F, Legendre P. Ward’s Hierarchical Agglomerative Clustering Method: Which Algorithms Implement Ward’s Criterion? *Journal of Classification*. 2014;31:274-295.

24. Zoppoli G, Regairaz M, Leo E, et al. Putative DNA/RNA helicase Schlafen-11 (SLFN11) sensitizes cancer cells to DNA-damaging agents. *Proc Natl Acad Sci U S A*. Sep 11 2012;109(37):15030-5. doi:10.1073/pnas.1205943109

Supplementary figures and figure legends

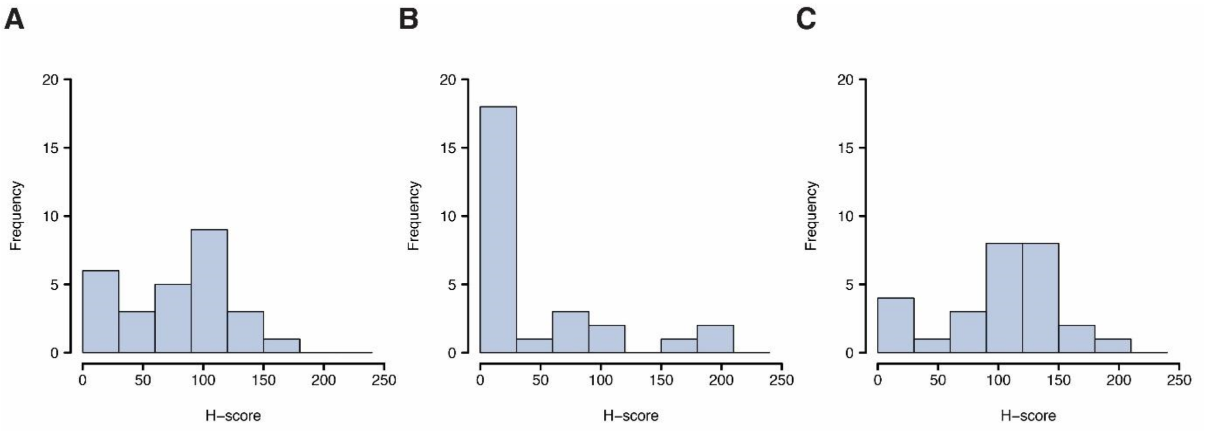

Figure S1: Frequency distribution of SLFN11 H-scores in the analyzed HGSOC case set. Nuclear SLFN11 protein in cancer and non-cancer cells was blindly assessed with automated image analysis by Halo and quantified as an H-score in all nuclear cells (A), cancer cells (B), and non-cancer cells (C). *y* axis: frequency of samples within each H-score bin; *x* axis: H-score values, subdivided into increasing 30-unit bins.

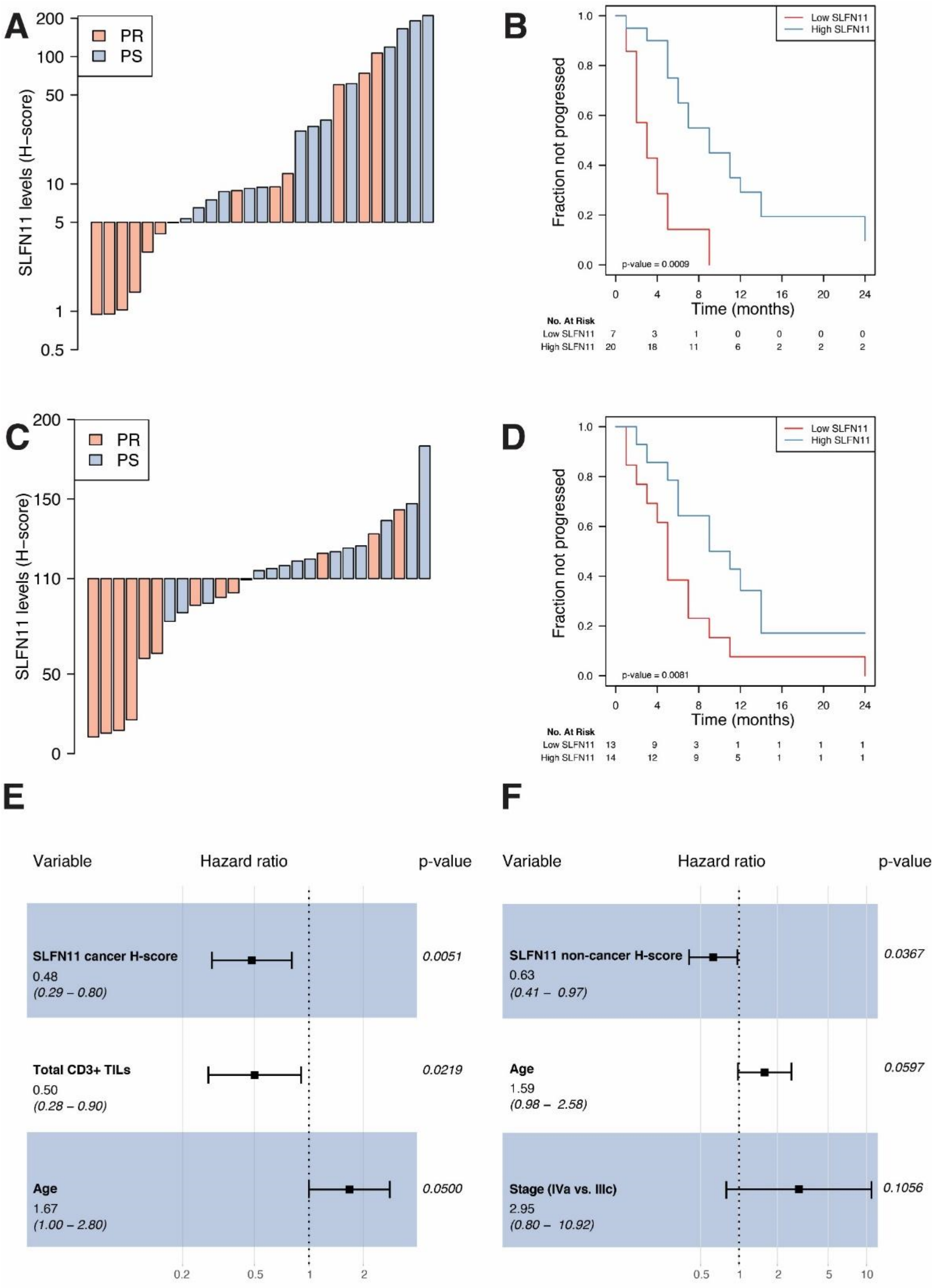

Figure S2: SLFN11 is independently prognostic in HGSOC also when assessed in cancer or non-cancer cells only. ***A and C:*** Waterfall plot showing SLFN11 protein levels in cancer (A) and non-cancer (C) cells, colored by platinum sensitivity: SLFN11 protein is reported as H-score (*y* axis), whereas cases are reported by increasing values (*x* axis) and colored in red if platinum-refractory (PR) or light blue if non-refractory (NR). ***B and D:*** Kaplan-Meier plots showing progression-free interval (PFI) stratified by SLFN11 cancer protein levels (“high” if H-score > 5, “low” if < 5, B) and

non-cancer protein levels (“high” if H-score > 110, “low” if < 110, B). The progressed fraction of patients (*y* axis) is plotted against time expressed in months from the end of first-line chemotherapy, censored at 24 months (*x* axis). Numbers at risk are reported below the plots. P-values in the bottom left of the plots are from the Wald statistics for the univariable Cox’s regression. ***E and F:*** Forest plots of hazard ratios (x axis, in log scale) for SLFN11 protein in cancer (E) and non-cancer (F) cells together with variables retained by the multivariable model generated for overall SLFN11. Point HR estimates are reported below each variable together with 95% confidence intervals (95%CI) in parentheses, whereas adjusted p-values for each variable are on the right side of the plot. Filled black squares represent HR estimates, with relative 95%CI shown as horizontal lines with brackets.

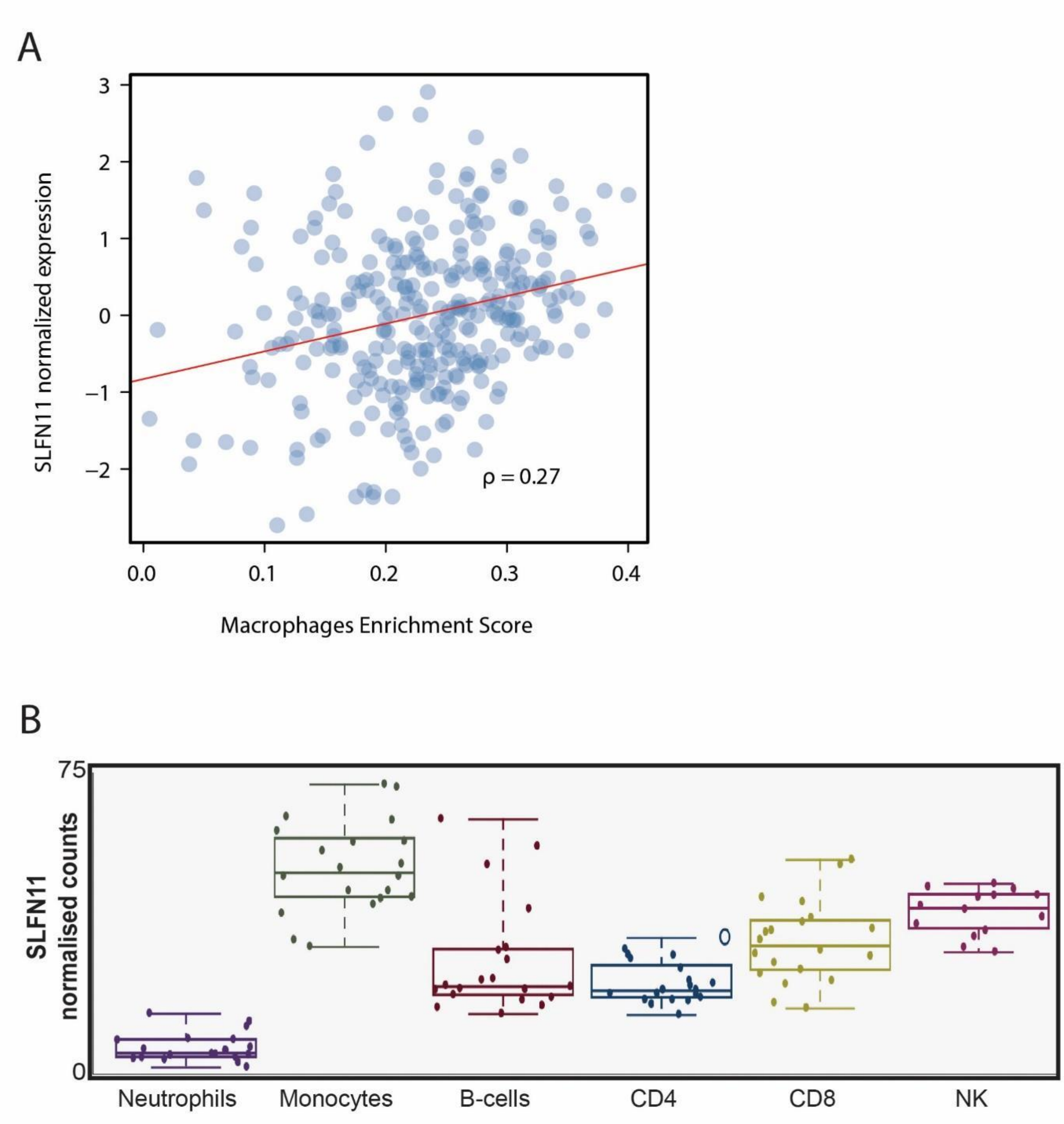

Figure S3: SLFN11 is expressed in a subset of leukocyte subpopulations. *A:* Scatterplot representing SLFN11 expression (log-transformed normalized counts, *y* axis) as a function of GSEA macrophage enrichment score (ES, *x* axis) in TCGA OVCAR dataset (N = 302); ρ is the Spearman's correlation coefficient, the least squares regression are represented by the red lines, whereas dots are measurements of SLFN11 expression by ES in individual samples. ***B:*** Box plots of publicly available RNA-sequencing results (GEO accession number GSE60424) for SLFN11 in sorted leukocyte subpopulations from patients. NK: natural killer cells. GEO: gene expression omnibus.

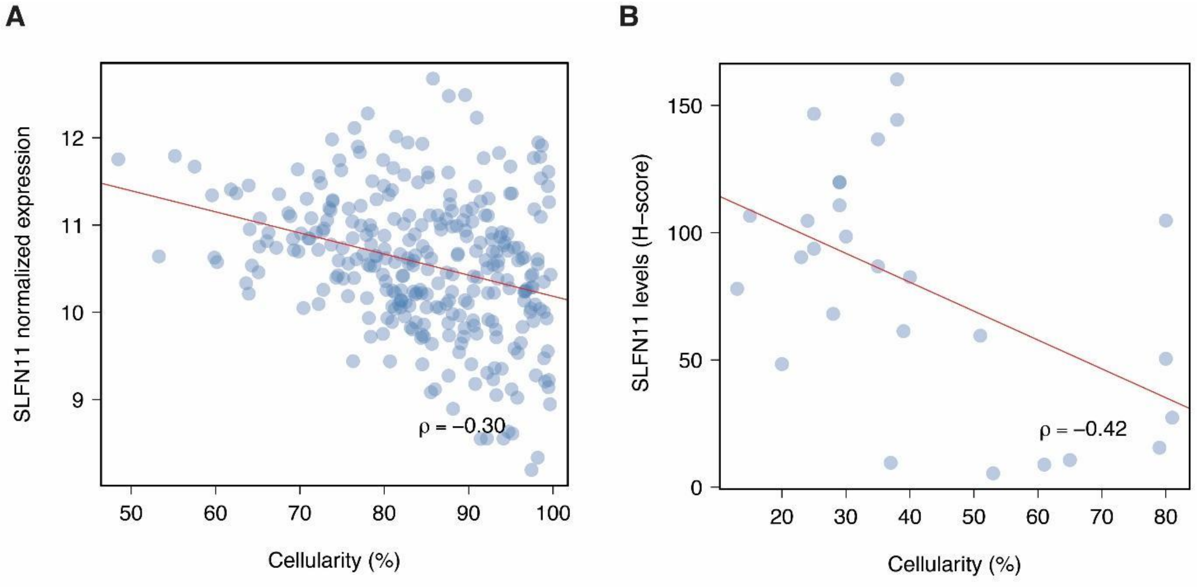

Figure S4: Cancer cellularity is negatively correlated with SLFN11 in ovarian cancer. ***A:*** Scatterplot representing SLFN11 transcript (log-transformed normalized counts, *y* axis) as a function of cancer cellularity inferred using ESTIMATE (*x* axis) in TCGA OVCAR dataset (N = 302). ***B:*** Scatterplot representing SLFN11 protein measured in all nuclear cells (overall H-score, *y* axis) as a function of cancer cellularity measured by HALO (*x* axis) in our cohort (N = 27). ρ is the Spearman's correlation coefficient, the least squares regression are represented by the red lines, whereas dots are measurements of SLFN11 protein levels by cancer cellularity in individual samples.

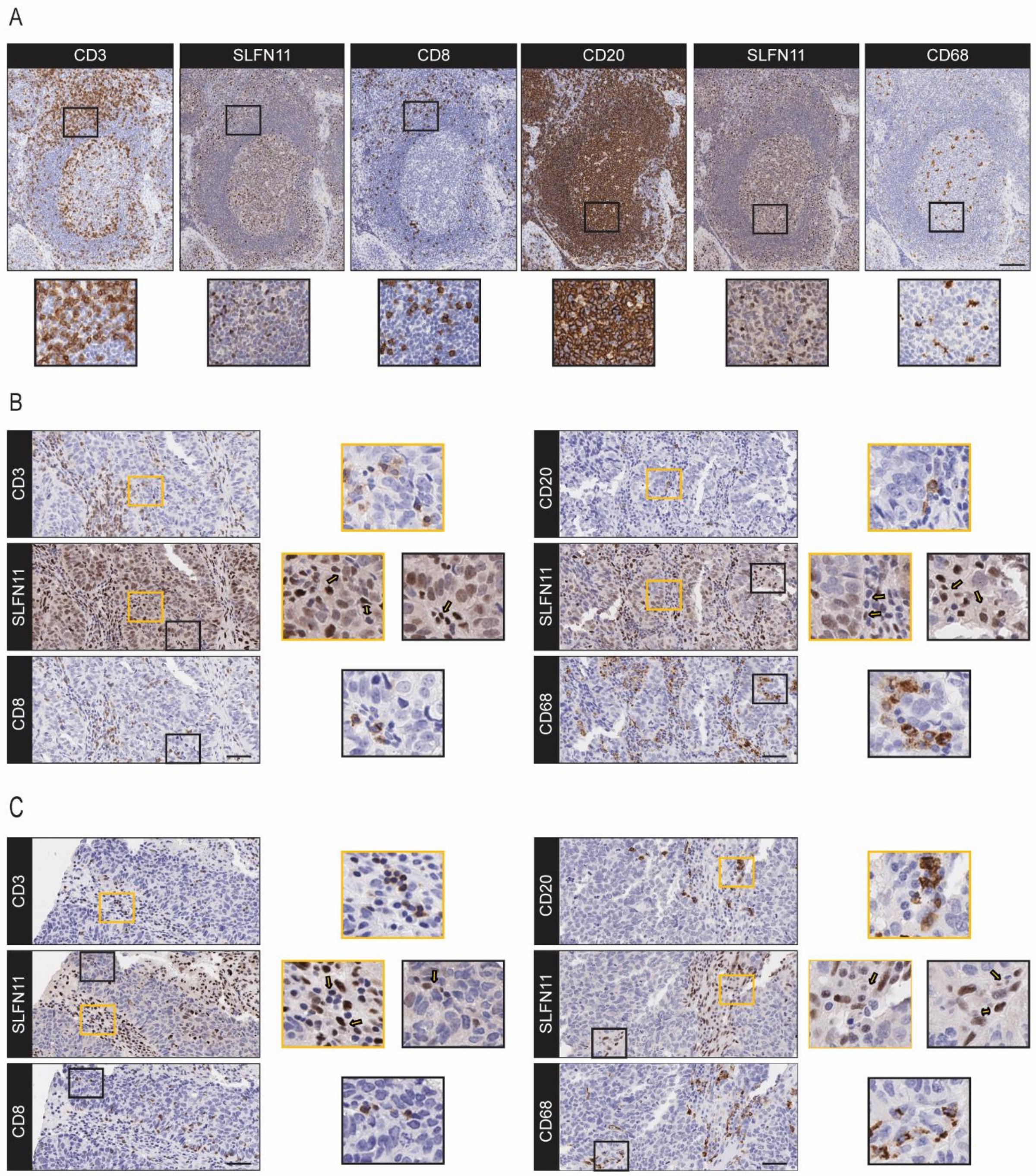

Figure S5: SLFN11 is expressed in a subset of immune-related cells in tonsil and HGSOC tissues. ***A:*** Representative images of CD3, CD8, SLFN11, CD20 and CD68 IHC on serial sections of tonsil tissue. Shown is a lymphoid follicle with the round- to oval shaped germinal center, the surrounding mantle zone and, at the outer layer of the lymphoid follicle, the paracortical zone. SLFN11 is mainly expressed in the germinal center, which is mostly composed of B-cells (CD20+) and macrophages/monocytes (CD68+), as well as in the T-cell rich (CD3+/CD8+)

paracortical zone. ***B and C:*** Representative images of CD3, CD8, SLFN11, CD20 and CD68 IHC on serial sections of tumor SLFN11 high- (B) and low (C) and stroma SLFN11 high cancers. The insets show nuclear SLFN11 in cytosolic/membrane-based CD3, CD8, CD20 and CD68 positive cells (indicated by arrows in B and C). Scale bars, 100 µm (A) and 50 µm (B). The insets show a 3x magnification of the representative images.

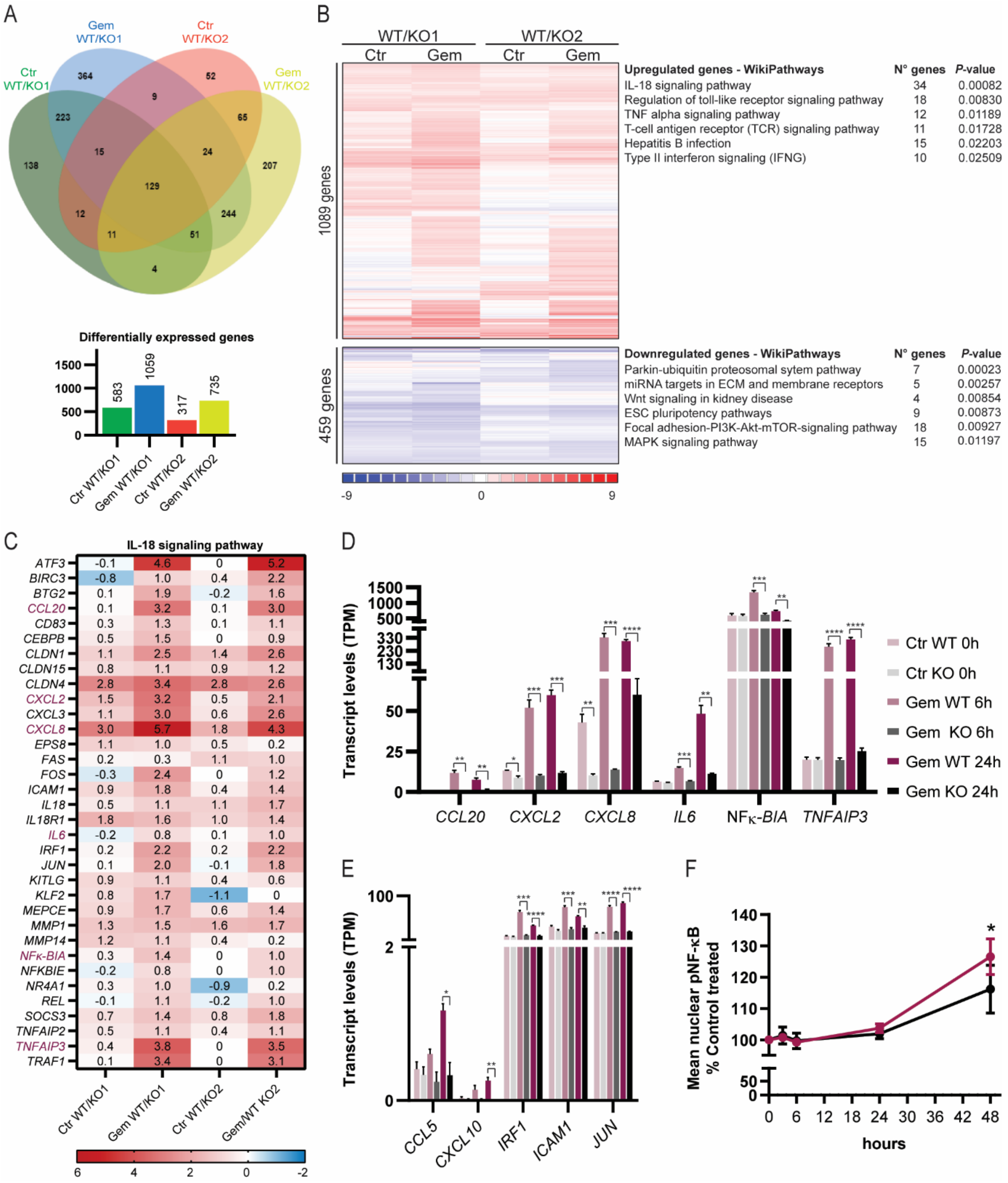

Figure S6: DNA damaging agent treatment induces the expression of immune-related genes in SLFN11-proficient cells. ***A:*** RNA-sequencing results of DU145 wild type (WT) and SLFN11-KO cells (2 different clones, KO1 and KO2) treated with control or gemcitabine for 6 hours as further described in the material and methods section. The Venn diagrams illustrate the overlap in significantly altered genes in control (Ctr) and gemcitabine (Gem) treated conditions (FDR<0.01 and 2-fold change) and the bar

chart below shows the differentially expressed gene numbers in the different comparisons. ***B:*** Meta-analysis of the RNA-sequencing datasets as indicated in panel A. The figure shows the heat map of the significantly up- (red shades) and down-regulated (blue shades) gene clusters (FDR<0.01 and 2-fold change, n=3) as well as their top canonical WikiPathways, as identified with OnRamp BioInformatics Rosalind genomic analysis platform. The list of significantly changed genes is available in supplementary table S7. ***C:*** Heat map of the up- (red shades) and down-regulated (blue shades) genes (log2 fold change) of the IL-18 signaling pathway shown in B in the indicated conditions. ***D, E:*** Bar charts of RNA-sequencing data showing the relative expression of some of the genes of the IL-18 (D) and Type II interferon signaling (IFNG) pathway (E) in WT or SLFN11 KO cells treated for 0, 6 or 24 hours with gemcitabine. *IRF1*, *ICAM1* and *JUN* gene are also part of the IL-18 signaling pathway (panel C). Data are presented as mean ±s.e.m. (n=3). **P*<0.05; ***P*<0.01; ****P*<0.001; *****P*<0.0001, (unpaired Student’s *t*-test, GraphPad Prism V8). ***F:*** pNFκ-B p65 Ser365 mean intensities in WT and SLFN11 KO cells treated for the indicated time points with gemcitabine as determined by immunofluorescence. The data are presented as mean±s.d. of the control-treated condition (n=3). **P*<0.05; ***P*<0.01, (paired Student’s *t*-test, GraphPad Prism V8).

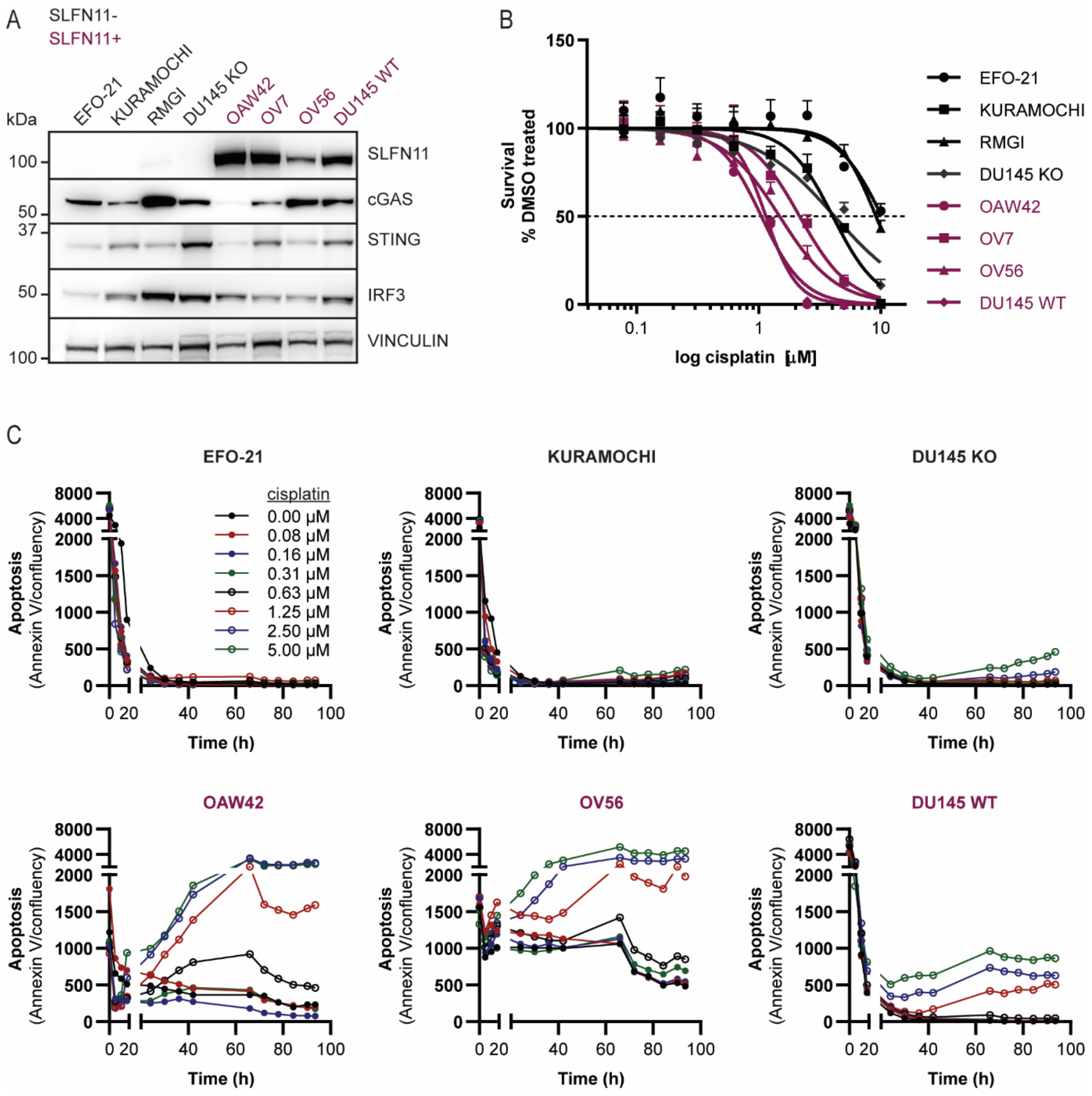

Figure S7: Response to cisplatin in SLFN11 proficient and deficient ovarian cancer cell lines. ***A:*** Representative immunoblots of the indicated proteins in 3 SLFN11-deficient (SLFN11-) and 3 SLFN11-proficient (SLFN11+) ovarian cancer cell lysates. The DU145 prostate cancer isogenic pair (WT and SLFN11 KO) served as control for SLFN11 expression. Vinculin served as loading control. ***B:*** Response to cisplatin in the indicated cell lines as determined by CellTiter-Glo luminescent viability assays (n=3). Data are presented as mean percentages ±s.d. of the control-treated conditions. IC_{50}: 10.2 (EFO-21); 4.0 (KURAMOCHI); 9.1 (RMGI); 4.2 (DU145 SLFN11 KO); 1.1 (OAW42); 2.2 (OV7); 1.5 (OV56); 1.1 (DU145 WT). ***C:*** Apoptosis of the indicated cell lines following cisplatin treatment as determined by annexin V staining. Apoptosis activity is represented by mean fluorescence levels of annexin V normalized

to total cell confluency. % confluency and fluorescence intensities were calculated using incucyte software.

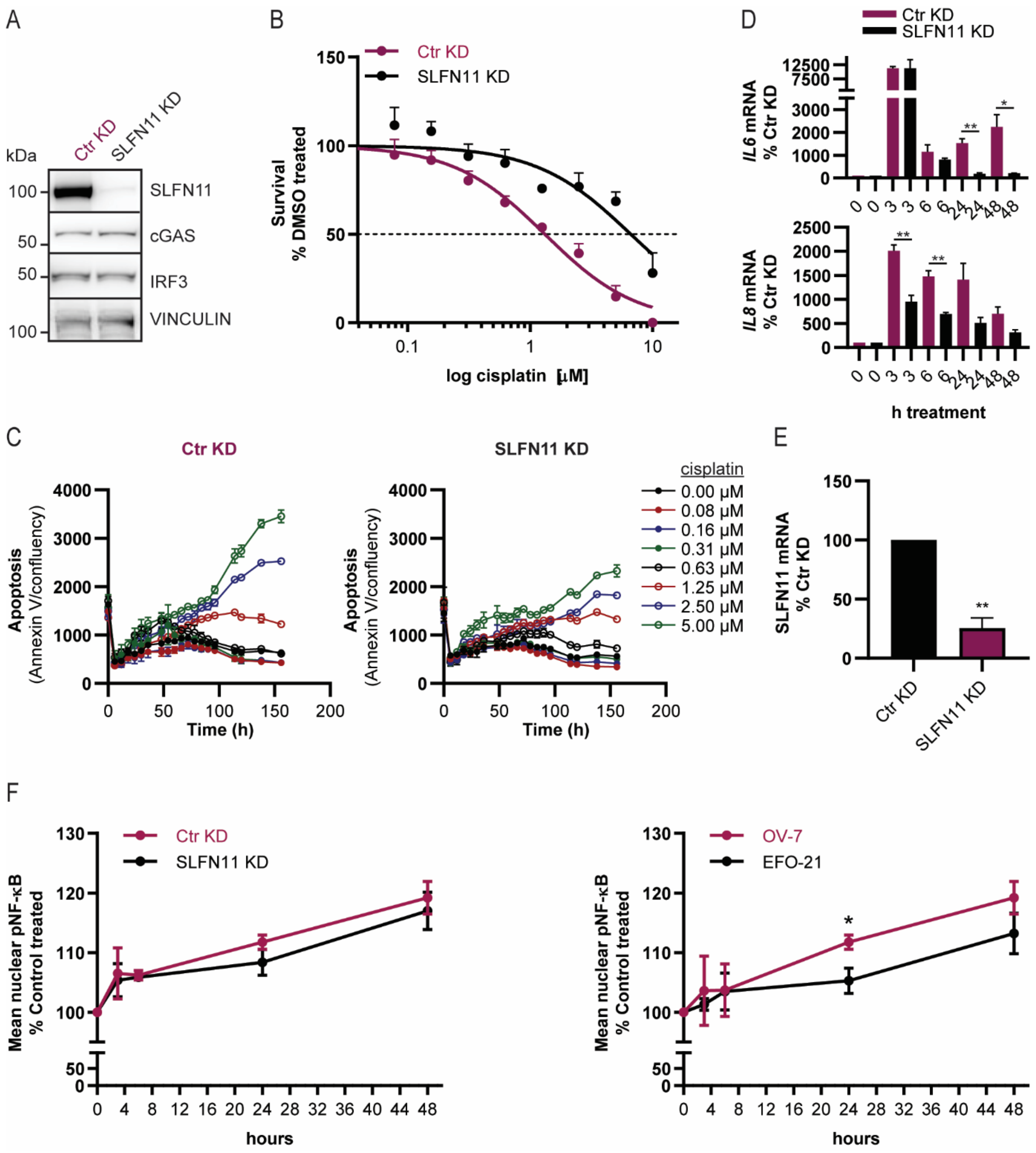

Figure S8: Cisplatin treatment activates an immune response in SLFN11-proficient OV-7 cells. ***A:*** Representative immunoblots of the indicated proteins in control KD and SLFN11 KD OV7 ovarian cancer cell lysates. Vinculin served as loading control. ***B:*** Response to cisplatin in the indicated cell lines as determined by CellTiter-Glo luminescent viability assays (n=3). Data are presented as mean percentages ±s.d. of the control KD condition. ***C:*** Apoptosis of the indicated cell lines following cisplatin treatment as determined by annexin V staining. Apoptosis activity is represented by mean fluorescence levels of annexin V to total cell confluency. % confluency and fluorescence intensities were calculated using incucyte software. ***D:***

mRNA levels of the indicated genes were measured by qRT-PCR in control and SLFN11 KD OV-7 cells treated for different time points with cisplatin. Data are represented as a mean±s.e.m. of the control KD treated condition (n=3). Similar results were obtained in a second independent experiment. ***E:*** SLFN11 mRNA expression in control KD and SLFN11 KD OV7 cells (n=3). Data are presented as mean percentages ±s.d. of the control KD condition. ***F:*** pNFκ-B p65 Ser365 mean intensities in control and SLFN11 KD OV-cells (left) and SLFN11-proficient OV-7 cells (same data set as in the left plot, caveat) versus SLFN11-deficient EFO-21 cells (right) treated for the indicated time points with cisplatin as determined by immunofluorescence. The data are presented as mean±s.d. of the control KD condition (n=2-3). *P<0.05; **P<0.01, (paired Student's *t*-test, GraphPad Prism V8).

A

Function	Pathway	Gene
Inhibitory (and Exhaustion)	PD1/PDL1	PDCD1
Inhibitory		CD274
Inhibitory	CTLA4/CD80	CTLA4
Inhibitory		CD80
Inhibitory		CD86
Inhibitory	B7-H3/CD276	CD276
Inhibitory	B7-H4/B7S1/B7x	VTCN1
Inhibitory	HVEM/BTLA	TNFRSF14
Inhibitory		BTLA
Inhibitory (and Exhaustion)	LAG3/CD223	LAG3
Inhibitory	GAL9/TIM3	LGALS9
Inhibitory (and Exhaustion)		HAVCR2
Inhibitory	IDO	IDO1
Inhibitory	CEACAM1/CD66	CEACAM1
Inhibitory	SIRP alpha/CD47	SIRPA
Inhibitory		CD47
Inhibitory	2B4/CD48	CD48

Function	Pathway	Gene
Co-stimulatory	CD266/CD155	PVR
Co-stimulatory	CD40	CD40
Co-stimulatory		CD40LG
Co-stimulatory	OX40/OX40L	TNFRSF4
Co-stimulatory		TNFSF4
Co-stimulatory	HVEM/LIGHT	TNFRSF14
Co-stimulatory		ADORA2A
Co-stimulatory	CD28/CD80	CD28
Co-stimulatory		CD80
Co-stimulatory	CD27/CD70	CD27
Co-stimulatory		CD70
Co-stimulatory	4-1BB/4-1BBL	TNFRSF9
Co-stimulatory		TNFSF9
Co-stimulatory	ICOS/ICOSL	ICOS
Co-stimulatory		ICOSLG

Function	Protein	Gene
Exhaustion	2B4	CD244
Exhaustion	CD160	CD160

B

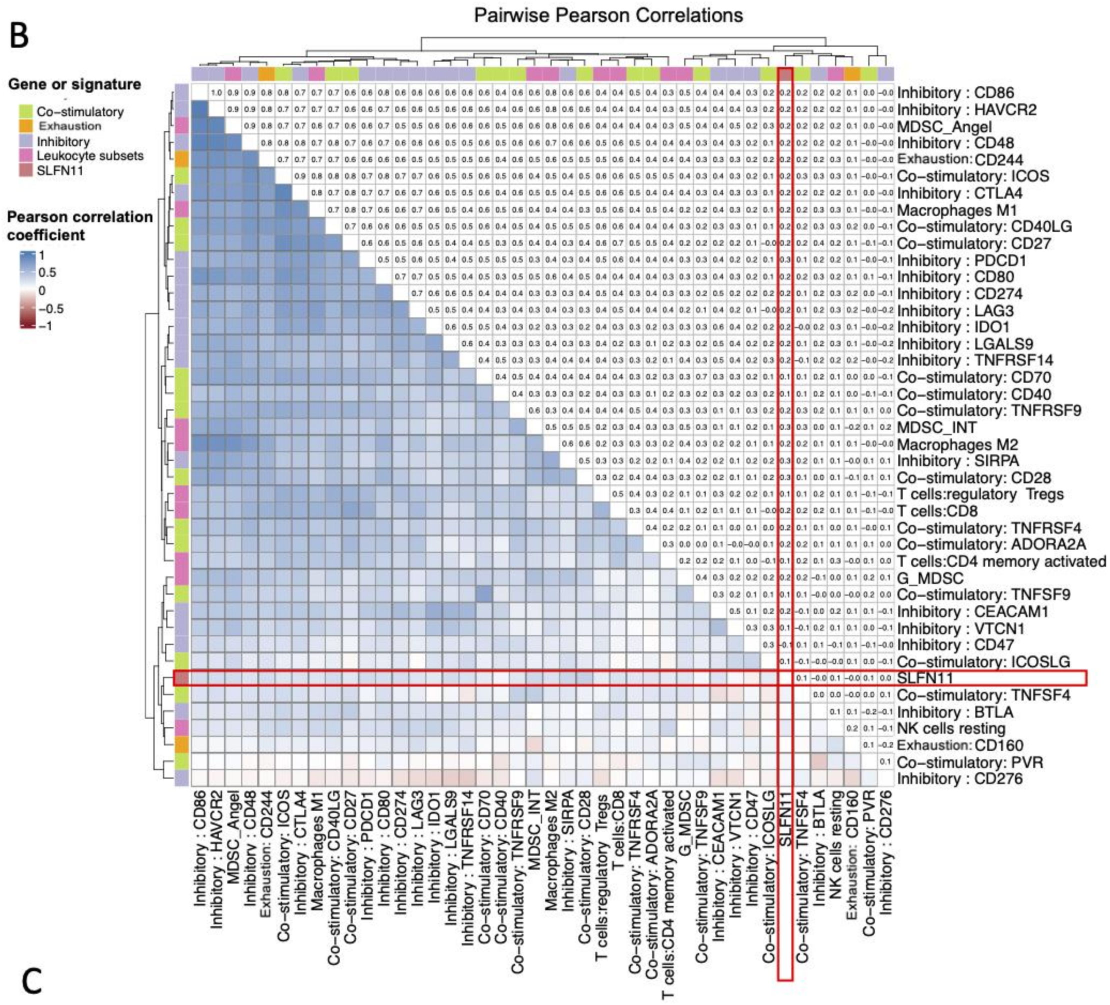

C

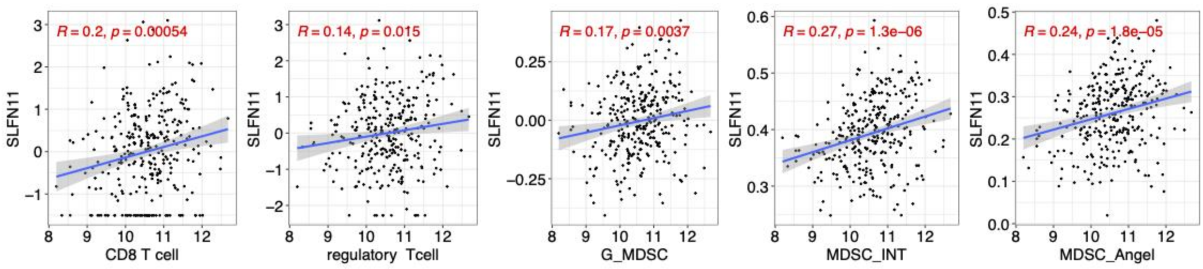

Figure S9: Correlation between SLFN11 and markers of suppression, exhaustion and immune activation in ovarian cancer. ***A:*** List of exhaustion

markers, co-stimulatory, and inhibitory checkpoint pathways assessed for correlation with SLFN11 in the ovarian cancer TCGA cohort (N=302). ***B:*** Heatmap matrix based on pairwise Pearson correlation coefficients between i) SLFN11 gene expression and ii) inhibitory, co-stimulatory, and exhaustion transcripts, as listed in panel A, iii) myeloid-derived suppressive cell (MDSC) enrichment scores (estimated using single sample GSEA, see methods), and regulatory T cells (estimated using CIBERSORTX). Five additional leucocyte subsets, estimated by CIBERSORTX, having the highest correlation with SLFN11 (see Table S5) are represented for contrast purposes to facilitate data interpretation. These include: M2 Macrophages, CD8 T cells, M1 Macrophages, NK cells resting, and activated CD4 memory T cells. ***C:*** Scatter plots displaying correlation between SLFN11 and CD8 T cells, regulatory T cells, and MDSC estimates. Pearson correlation was used in view of the normal/quasi-normal distribution of most the analyzed markers.
